# From promise to practice: insights into ChatGPT-4o use in child and adolescent mental health from professionals

**DOI:** 10.3389/fpsyt.2025.1668814

**Published:** 2025-09-26

**Authors:** Armagan Aral, Gizem Gerdan, Mirac Barıs Usta, Ayse Erguner Aral

**Affiliations:** 1 Department of Child and Adolescent Psychiatry, City Hospital of Izmır, Izmır, Türkiye; 2 Department of Psychology, Division of Clinical Psychology, Izmır Democracy University, Izmır, Türkiye; 3 Department of Child and Adolescent Psychiatry, Ondokuz Mayıs University, Faculty of Medicine, Samsun, Türkiye; 4 Department of Psychiatry, City Hospital of Izmır, Izmır, Türkiye

**Keywords:** ChatGPT-4o, child and adolescent, clinical integration, mental health, professional perspectives

## Abstract

**Background:**

Despite growing interest, empirical studies on ChatGPT-4o’s clinical role in child and adolescent mental health remain scarce. This study explored child and adolescent mental health professionals’ attitudes toward ChatGPT-4o, focusing on its clinical applications, ethical implications, and integration challenges.

**Methods:**

A sequential exploratory design was used, beginning with interviews to inform item generation. Finalized surveys were distributed online to 96 child and adolescent psychiatrists and 70 psychologists between April and May 2025. The instrument measured views across seven subscales and demonstrated strong internal consistency (α = 0.887 for child and adolescent psychiatrists; α = 0.903 for psychologists).

**Results:**

Overall, 47.9% of psychiatrists and 40% of psychologists reported prior use of ChatGPT-4o. Child and adolescent psychiatrists rated “Clinician-Facing Tool” and “Acting as a Therapist” most favorably, while psychologists expressed the most positive views toward “Bias” and “Profession”. Both groups viewed “Ethical Issues” least favorably. Comparative analyses revealed that psychiatrists scored significantly higher than psychologists on the profession (*d* = 0.46), psychoeducational use within treatment (*d* = 0.43), patient-facing tool (*d* = 0.68), digital access and personalization (*d* = 0.55), and crisis prevention and safety planning (*d* = 0.69). Psychiatrists also showed greater positive views toward self-help and behavior change interventions (U = 2649.5, Z = –2.41, *p* = 0.016, *r* = 0.19). In contrast, psychologists rated bias more favorably, representing the largest observed difference (*d* = 1.56). Development priorities differed slightly: child and adolescent psychiatrists emphasized software support for diagnostic & treatment, system oversight, and ethics, while psychologists also prioritized system oversight and ethics.

**Conclusion:**

Mental health professionals show cautious optimism toward ChatGPT-4o, with discipline-specific emphases. While a measured interest in ChatGPT-4o’s clinical integration, shared concerns around ethics highlight the need for role-specific guidelines and human oversight.

## Graphical Abstract

**Figure:**
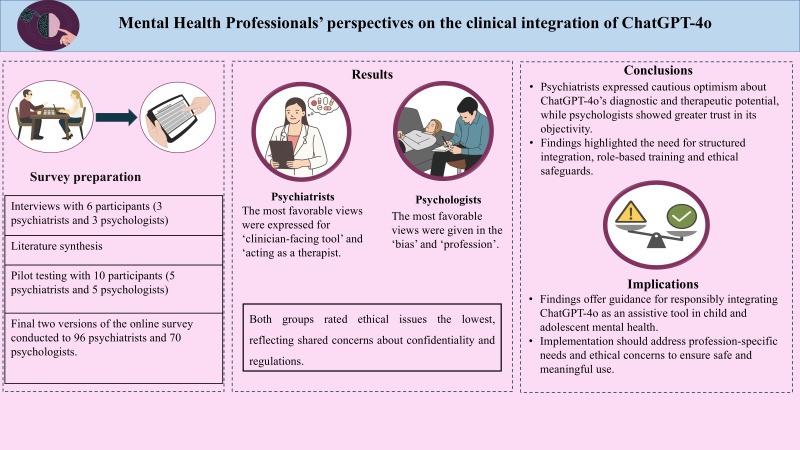

## 1 Introduction

ChatGPT has rapidly drawn attention for its medical applications, including mental health. It enables fast, context-aware responses and is being explored for use in mental health due to its ability to synthesize clinical data and offer supportive responses. With mental health service demand outpacing supply, such tools may help streamline care tasks (1). Still, strong empirical evidence on safety and efficacy is lacking (2), and early evaluations urge caution in high-stakes settings (3). Given the complexity of psychiatric disorders, concerns persist about appropriate use, patient guidance, and clinical standards.

Most peer-reviewed literature on ChatGPT in mental health comprises reviews and commentaries (4, 5), with limited clinician input often shaped by prompt-based designs and researcher familiarity, reducing external validity (6). Although scholarly interest is rising, there has yet to be a systematic exploration of how child and adolescent mental health professionals view ChatGPT integration in clinical practice. Existing investigations have largely centered on general mental health via surveys (3). While some researches provides useful insights into how adult psychiatrists in Turkey perceive ChatGPT, it focuses primarily on general awareness and usage patterns through a quantitative lens (7). In contrast, the present study centers on mental health professionals—specifically child and adolescent psychiatrists and psychologists—offering a more specialized perspective on the ChatGPT-4o’s role in this sensitive population. By analyzing quantitative responses across key subdimensions such as clinician-facing, patient-facing, and acting as a therapist, our research provides in-depth and practice-oriented insights.

In June 2023, the APA issued a cautionary stance on the unregulated use of chatbots (8), while formal guidance from other professional bodies remains limited or under development. Notably, there is currently no global consensus regarding the integration of AI tools like ChatGPT in child and adolescent mental health care, echoing broader concerns in the literature that emphasize fragmented and insufficient international guidelines on AI in healthcare (9). This lack of unified standards reflects broader uncertainties around safety, accountability, and developmental appropriateness. As such, clinicians often rely on personal judgment in navigating ethical and practical considerations. Given the nascent stage of AI (Artificial Intelligence) integration in Turkey’s mental health system (10), early clinician feedback is crucial for shaping its role in child and adolescent mental health care. Therefore, the present study surveyed the perspectives of child and adolescent mental health professionals on the role of ChatGPT-4o in clinical practice, offering a unique exploration of its benefits, ethical risks, and practical limitations. As tools like ChatGPT-4o become increasingly embedded in clinical workflows, the reflections of these professionals on its expected opportunities and obstacles can inform more effective mental health service strategies.

## 2 Materials and methods

### 2.1 Study design and setting

This study employed a quantitative descriptive design and was conducted between April and May 2025. Two online surveys were developed via Google Docs—one designed for child and adolescent psychiatrists, the other for psychologists. Item structures followed recent guidance for adapting AI tools to clinical populations (11). To maintain data integrity, each participant was permitted to complete the survey only once. The City Hospital of Izmır Ethical Committee regarding non-interventional clinical research reviewed and approved the study on March 19, 2025 (No: 2025/142).

### 2.2 Participants and recruitment

A total of 96 child and adolescent psychiatrists and 70 psychologists working with children and adolescents currently practicing in Turkey participated in the study. Recruitment was conducted using purposive and convenience sampling through professional WhatsApp groups and peer-to-peer referrals. This recruitment strategy mirrors recent digital mental health studies that utilized clinician networks for distributing AI-related surveys (3). Only licensed professionals were eligible to participate, and no financial incentives were provided. Given the absence of an official national registry, the precise size of the population of child and adolescent psychiatrists and psychologists practicing in Turkey remains uncertain. Our sample thus reflects a portion of this professional community, recruited via convenience and purposive methods, rather than a census of the entire population. Consequently, findings should be interpreted as exploratory and not assumed to represent all practitioners.

### 2.3 Interview process and survey development

Prior to survey construction, in-depth interviews were conducted with 3 child and adolescent psychiatrists and 3 psychologists to explore their perceptions regarding the clinical use of ChatGPT-4o. These interviews, conducted in Turkish, either online or in person at a child and adolescent psychiatry clinic, lasted between 40 and 55 minutes (M = 46). Verbatim transcripts were reviewed and verified by participants; anonymization was ensured using coded identifiers (e.g., PSY1–3 for child and adolescent psychiatrists, PSL1–3 for psychologists). 6 interview prompts (translated in English) and identifier coding details can be found in **Supplementary Material 1**. This qualitative phase served to inform item generation, consistent with methods employed in comparable LLM (Large Language Model)-related studies (7). Instead of formal thematic analysis, items were derived directly from expert input and targeted literature synthesis. This approach is supported in the development of pragmatic instruments for clinical populations (12, 13). Two preliminary survey versions, tailored for child and adolescent psychiatrists and psychologists, were finalized and are presented in **Supplementary Material 2**.

### 2.4 Pilot testing

To assess item clarity and face validity, two pilot studies were conducted; one with 5 child and adolescent psychiatrists and one with 5 psychologists. Participants provided feedback on item phrasing, survey layout, and logical sequencing. Modifications were implemented accordingly to improve clarity and usability. A summary of feedback and revisions is provided in **Supplementary Material 3**. Final surveys were also reviewed using a think-aloud protocol and were designed to be completed in under eight minutes.

### 2.5 Survey structure

The finalized questionnaires were structured around seven conceptually distinct subscales (1): Profession (2), Ethical Issues, (3) Bias, (4) Clinician-Facing Tool, (5) Patient-Facing Tool, (6) Acting as a Therapist, and (7) General Impressions. Each subscale included multiple items, some of which were further grouped into subsections. For example, the “Clinician-Facing Tool” subscale comprised “Clinical Diagnosis,” “Treatment,” and “Documentation and Case Formulation”. Items were rated on a 5-point Likert scale. The full item set and coding schema are available in **Supplementary Material 4**. The structure was based on established instruments assessing clinician attitudes toward AI and was adapted for ChatGPT-4o’s role in psychiatric contexts (14).

### 2.6 Psychometric assessment

To evaluate the internal consistency of the instrument, Cronbach’s alpha coefficients were calculated for each subscale and for the overall scale. All analyses were conducted separately for child and adolescent psychiatrists and psychologists using coded items. A detailed breakdown of reliability scores and classification thresholds is provided in **Supplementary Material 4**.

### 2.7 Statistical analysis

Descriptive statistics were computed using SPSS version 25.0 to summarize participants’ responses across subscales. Frequencies, means, and standard deviations were calculated for all survey items. Internal consistency was assessed using Cronbach’s alpha to ensure the reliability of each subscale.

## 3 Results

**Figure 1:**
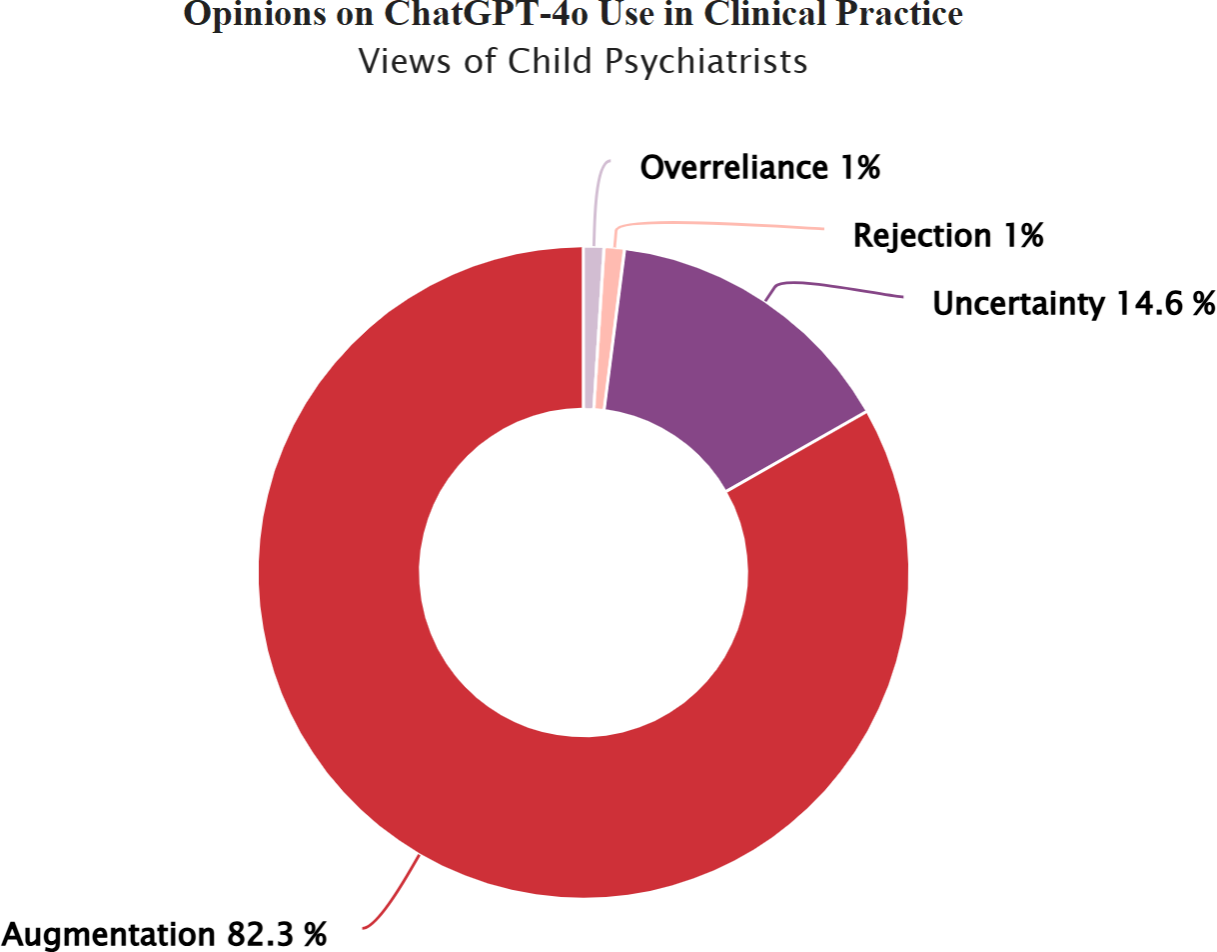
Child psychiatrists’ views on integrating ChatGPT-4o into clinical practice. Child psychiatrists’ views on integrating ChatGPT-4o into clinical practice. Response categories reflect varying perspectives on the integration of ChatGPT-4o in child and adolescent mental health practice: Rejection: ChatGPT-4o has no place in clinical practice, Overreliance: Unquestioning trust in ChatGPT-4o’s diagnostic and treatment suggestions, Uncertainty: Uncertainty regarding its clinical usefulness, Augmentation: A synergistic effect could emerge by combining mental health professionals' clinical expertise with ChatGPT-4o’s analytical capabilities.

**Figure 2:**
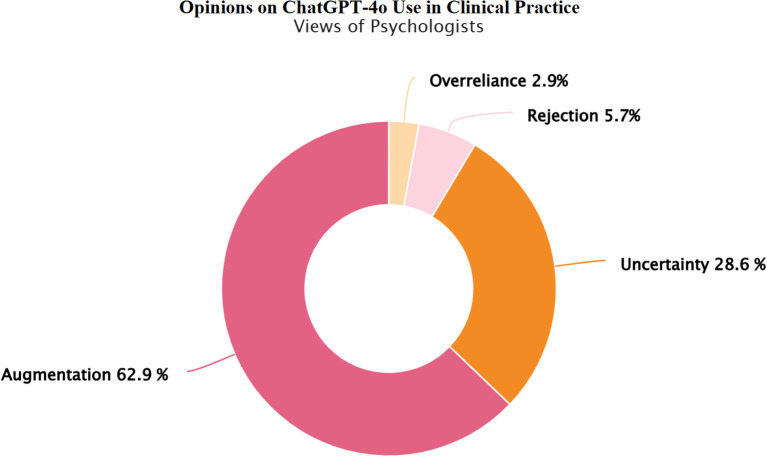
Psychologists’ views on integrating ChatGPT-4o into clinical practice. Psychologists’ views on integrating ChatGPT-4o into clinical practice. Response categories reflect varying perspectives on the integration of ChatGPT-4o in child and adolescent mental health practice: Rejection: ChatGPT-4o has no place in clinical practice, Overreliance: Unquestioning trust in ChatGPT-4o’s diagnostic and treatment suggestions, Uncertainty: Uncertainty regarding its clinical usefulness, Augmentation: A synergistic effect could emerge by combining mental health professionals' clinical expertise with ChatGPT-4o’s analytical capabilities.

The study included a total of 166 licensed professionals working in the field of child and adolescent mental health in Turkey. Demographic and usage characteristics of the participants, including age, gender, institutional affiliation, and prior ChatGPT-4o experience, are detailed in **Supplementary Material 5**. **Figures 1**, **2** further illustrate child and adolescent psychiatrists’ and psychologists’ initial opinions on integrating ChatGPT-4o into clinical practice. While a notable proportion in both groups endorsed its potential for augmenting clinical reasoning, psychiatrists exhibited relatively higher confidence in synergistic human-AI collaboration, whereas psychologists demonstrated a greater degree of uncertainty and skepticism. Taken together, these demographic patterns and initial opinions suggest both shared and discipline-specific approaches in how mental health professionals interact with ChatGPT-4o.

To further contextualize these initial opinion patterns, all items coded from A1 to G5, reflecting clinician perspectives on the role of ChatGPT-4o in mental health practice, were measured using a 5-point Likert scale (1 = Strongly Disagree to 5 = Strongly Agree). By analyzing mean scores rather than relying solely on categorical breakdowns, the study was able to highlight more subtle differences in how participants perceived the clinical use of ChatGPT-4o insights that might be overlooked with a simple agree/disagree format (15). For interpretive consistency, higher scores were coded to reflect more favorable views toward ChatGPT-4o integration in clinical practice. Mean scores were interpreted based on defined intervals, where values between 2.60 and 3.39 were classified as indicating a neutral view, scores below 2.60 reflected negative views, and scores above 3.40 indicated moderately to strongly positive views. The complete interpretation framework used for this classification is provided in **Supplementary Material 4**, **Supplementary Table S4.3**. Reverse-coded items were adjusted accordingly to ensure that mean values consistently represented the direction of positive views, and all scoring procedures are documented in **Supplementary Material 4**.

The following tables function as interpretive tools that capture how child and adolescent mental health professionals in Turkey evaluate the clinical integration, perceived benefits, and considerations surrounding ChatGPT-4o within mental health practice. **Table 1** shows that child and adolescent psychiatrists most strongly endorsed the “Clinician-Facing Tool” subscale (M = 3.79, SD = 0.47), especially valuing its diagnostic utility (Clinical Diagnosis) (M = 3.88, SD = 0.51). Following this, the second-highest scoring domain was “Acting as a Therapist” (M = 3.69, SD = 0.65). In particular, the “Self-Help and Behavior Change Interventions” subsection was rated favorably (M = 3.75, SD = 0.74). “Ethical Issues” received the lowest mean (M = 1.92). A similar but more reserved pattern emerged among psychologists (**Table 2**). Only “Bias” (M = 3.75) and “Profession” (M = 3.40) reached the high range. As in the psychiatrist group, the Ethical Issues subscale received the lowest average score (M = 2.46, SD = 0.91). Nevertheless, both groups showed strong internal consistency for the full instrument (α = 0.887^G^ for psychiatrists; α = 0.903^E^ for psychologists).

**Table 1:** Child and adolescent psychiatrists’ mean scores (M ± SD) on items regarding the views on the role of ChatGPT-4o in clinical practice (N = 96).

Subscale	Subsection	Subsection theme	Items	Mean ± SD	Cronbach’s α
Profession			*A1*	1.69 ± 0.73	
			*A2*	4.05 ± 0.82	
			*A3*	3.99 ± 0.90	
			*A4*	4.25 ± 0.79	
			*A5*	4.39 ± 0.71	
			**Subscale Mean**	**3.67 ± 0.58**	**α = 0.781^A^**
Ethical Issues			*B1*	2.30 ± 1.03	
			*B2*	1.54 ± 0.80	
			**Subscale Mean**	**1.92 ± 0.75**	***^na^***
As a Clinician-Facing Tool	**Documentation and Case Formulation**		*C1*	2.98 ± 1.06	
			*C2*	3.81 ± 0.73	
			*C3*	4.00 ± 0.66	
			*C4*	4.09 ± 0.65	
			*C5*	3.91 ± 0.75	
			*C6*	3.45 ± 0.86	
			**Subsection Mean**	**3.70 ± 0.54**	
	**Clinical Diagnosis**		*C7*	3.98 ± 0.66	
			*C8*	3.67 ± 0.90	
			*C9*	3.97 ± 0.67	
			**Subsection Mean**	**3.88 ± 0.51**	
	**Treatment**	**Psychoeducation**	*C10*	3.47 ± 0.95	
		**Management**	*C11*	3.82 ± 0.83	
		**Medication Guidance**	*C12*	3.55 ± 0.89	
			*C13*	4.00 ± 0.76	
			*C14*	3.94 ± 0.84	
			**Subsection Theme Mean**	**3.82 ± 0.73**	
		**Structured Psychotherapeutic Support**	*C15*	3.68 ± 0.90	
			*C16*	3.65 ± 0.93	
			*C17*	3.85 ± 0.78	
			*C18*	3.95 ± 0.71	
			**Subsection Theme Mean**	**3.78 ± 0.73**	
			**Subsection Mean**	**3.75 ± 0.64**	
			**Subscale Mean**	**3.79 ± 0.47**	**α = 0.727^A^**
As a Patient-Facing Tool	**Risks of Independent Use**		*D1*	2.81 ± 1.12	
	**Psychoeducational Support for Families**		*D2*	3.69 ± 0.81	
			*D3*	3.27 ± 0.92	
			*D4*	3.46 ± 0.91	
			**Subsection Mean**	**3.47 ± 0.73**	
	**Digital Access and Personalization in Youth Mental Health**		*D5*	3.55 ± 0.93	
			*D6*	3.25 ± 0.97	
			*D7*	3.87 ± 0.88	
			**Subsection Mean**	**3.55 ± 0.72**	
			**Subscale Mean**	**3.41 ± 0.58**	**α = 0.727^A^**
Acting as a Therapist	**Therapeutic Alliance**		*E1*	3.34 ± 1.01	
	**Self-Help and Behavior Change Interventions**		*E2*	3.78 ± 0.86	
			*E3*	3.71 ± 0.89	
			*E4*	3.68 ± 0.84	
			*E5*	3.86 ± 0.85	
			*E6*	3.73 ± 0.82	
			**Subsection Mean**	**3.75 ± 0.74**	
	**Crisis Prevention and Safety Planning**		*E7*	3.54 ± 1.01	
			**Subscale Mean**	**3.69 ± 0.65**	**α =0.833^G^**
Bias			*F1*	**2.29 ± 0.93**	***^na^***
General Impressions			*G1*	3.77 ± 0.71	
			*G2*	3.58 ± 0.87	
			*G3*	3.38 ± 0.89	
			*G4*	2.47 ± 0.99	
			**Subscale Mean**	**3.29 ± 0.55**	**α =0.491^X^**
Willingness to Use ChatGPT-4o			*H. How willing are you to use ChatGPT-4o in your clinical practice?*	6.63 ± 2.24	
AI Development Priorities			*Ethical*	8.51 ± 1.93	
			*System Oversight*	8.82 ± 1.67	
			*AI Training for Professionals*	8.36 ± 2.13	
			*Software – Diagnostic & Treatment Support*	8.93 ± 1.66	
			*Software – Psychotherapy Support*	8.39 ± 2.21	

Overall scale internal consistency across all items: Cronbach’s α = 0.887^G^. **^E^**Excellent (α ≥.90) |**^G^**Good (.80–.89) |**^A^**Acceptable (.70–.79)|**^ᵠ^**Questionable (.60–.69) |**^x^**Poor (<.60)|**^na^**Not applicable (e.g., subscales with one or two items). Classification adapted from: Tavakol, M., & Dennick, R. (2011) (33).

Bolded values indicate Cronbach’s alpha coefficients for the corresponding subscales.

**Table 2:** Psychologists mean scores (M ± SD) on items regarding the views on the role of ChatGPT-4o in clinical practice (N = 70).

Subscale	Subsection	Subsection theme	Items	Mean ± SD	Cronbach’s α
Profession			*A1*	1.81 ± 1.14	
			*A2*	3.83 ± 0.91	
			*A3*	3.61 ± 0.90	
			*A4*	3.83 ± 0.88	
			*A5*	3.94 ± 0.77	
			**Subscale Mean**	**3.40 ± 0.58**	**α =0.608*^ᵠ^***
Ethical Issues			*B1*	2.01 ± 1.07	
			*B2*	2.96 ± 1.09	
			**Subscale Mean**	**2.46 ± 0.91**	***^na^***
As a Clinician-Facing Tool	**Documentation and Case Formulation**		*C1*	3.03 ± 1.02	
			*C2*	2.30 ± 0.89	
			*C3*	3.44 ± 0.91	
			*C4*	3.64 ± 0.88	
			*C5*	3.71 ± 0.90	
			*C6*	3.46 ± 0.97	
			*C7*	3.04 ± 1.08	
			**Subsection mean**	**3.23 ± 0.62**	
	**Clinical Diagnosis**		*C8*	3.44 ± 1.05	
			*C9*	3.00 ± 1.06	
			*C10*	3.57 ± 0.86	
			*C11*	2.69 ± 1.00	
			*C12*	2.13 ± 0.96	
			**Subsection mean**	**2.96 ± 0.59**	
	**Perceived Dehumanization**		*C13*	2.09 ± 0.96	
	**Treatment**	**Psychoeducation**	*C14*	3.01 ± 1.17	
		**Structured Psychotherapeutic Support**	*C15*	3.61 ± 0.98	
			*C16*	3.46 ± 1.12	
			*C17*	3.61 ± 1.02	
			*C18*	3.80 ± 0.95	
			**Subsection Theme Mean**	**3.62 ± 0.89**	
			**Subsection Mean**	**3.50 ± 0.84**	
			**Subscale Mean**	**3.16 ± 0.53**	**α =0.861^G^**
As a Patient-Facing Tool	**Psychoeducational Support for Families**		*D1*	3.47 ± 1.01	
			*D2*	2.20 ± 1.03	
			*D3*	2.86 ± 1.05	
			*D4*	2.90 ± 1.03	
			**Subsection Mean**	**2.85 ± 0.72**	
	**Digital Access and Personalization in Youth Mental Health**		*D5*	3.04 ± 1.01	
			*D6*	2.70 ± 1.14	
			*D7*	3.63 ± 1.14	
			**Subsection Mean**	**3.12 ± 0.83**	
			**Subscale Mean**	**2.97 ± 0.70**	**α =0.789^A^**
Acting as a Therapist	**Therapeutic Alliance**		*E1*	2.97 ± 1.25	
			*E2*	2.40 ± 1.21	
			**Subsection Mean**	**2.69 ± 1.09**	
	**Self-Help and Behavior Change Interventions**		*E3*	3.52 ± 0.97	
			*E4*	3.27 ± 1.12	
			*E5*	3.30 ± 1.20	
			*E6*	3.66 ± 0.94	
			*E7*	3.43 ± 1.10	
			**Subsection Mean**	**3.47 ± 0.88**	
	**Crisis Prevention and Safety Planning**		*E8*	2.75 ± 1.24	
			**Subscale Mean**	**3.20 ± 0.84**	**α =0.577^X^**
Bias			*F1*	**3.75 ± 0.93**	***^na^***
General Impressions			*G1*	3.09 ± 0.91	
			*G2*	3.31 ± 0.91	
			*G3*	3.34 ± 0.88	
			*G4*	2.99 ± 1.05	
			*G5*	2.50 ± 1.07	
			**Subscale mean**	**3.04 ± 0.56**	**α =0.512^X^**
Willingness to Use ChatGPT-4o			*H. How willing are you to use ChatGPT-4o in your clinical practice?*	5.94 ± 1.84	
AI Development Priorities			Ethical	8.50 ± 1.98	
			System Oversight	8.40 ± 2.22	
			AI Training for Professionals	8.27 ± 2.14	
			Software – Diagnostic & Treatment Support	8.27 ± 2.50	
			Software – Psychotherapy Support	7.81 ± 2.63	

Overall scale internal consistency across all items: Cronbach’s α = 0.903^E^. **^E^**Excellent (α ≥.90) |**^G^**Good (.80–.89) |**^A^**Acceptable (.70–.79)|**^ᵠ^**Questionable (.60–.69) |**^x^**Poor (<.60)|**^na^**Not applicable (e.g., subscales with one or two items). Classification adapted from: Tavakol, M., & Dennick, R. (2011) (33).

Bolded values indicate Cronbach’s alpha coefficients for the corresponding subscales.

Building on these descriptive findings, we next examined potential group differences across child and adolescent psychiatrists and psychologists. Comparisons between child and adolescent psychiatrists and psychologists are reported in **Table 3**. Because some subscales were not structurally equivalent across professions, analyses were limited to dimensions with parallel item structures. Where distributional assumptions were satisfied, independent-samples t-tests were applied; in other cases, Mann–Whitney U tests were employed. Child and adolescent psychiatrists reported higher scores on the professional role dimension (t (164) = –2.92, p = 0.004, d = 0.46) and on psychoeducational use within treatment (t(164) = –2.73, p = 0.007, d = 0.43). Similarly, child and adolescent psychiatrists evaluated patient-facing functions more favorably, both in the overall subscale (t(164) = –4.43, p < 0.001, d = 0.68) and in digital access and personalization (t(164) = –3.59, p < 0.001, d = 0.55). For therapeutic applications, child and adolescent psychiatrists placed stronger emphasis on crisis prevention and safety planning (t(164) = –4.47, p < 0.001, d = 0.69) as well as on self-help and behavior change interventions (U = 2649.5, Z = –2.41, p = 0.016, r = –0.19). In contrast, no group differences emerged for structured psychotherapeutic support (U = 3173.5, Z = –0.62, p = 0.534). Child and adolescent psychiatrists, however, expressed greater concern regarding potential bias compared to psychologists (t(164) = 9.74, p < 0.001, d = 1.56). The small, medium, and large effect sizes for the independent samples t-test (Cohen’s d) are considered to be 0.02, 0.05, and 0.08, respectively (16), whereas benchmarks for effect size *r* in Mann–Whitney U analyses are conventionally set at 0.10, 0.30, and 0.50, indicating small, medium, and large effects (17). Taken together, the results point to discipline-specific orientations toward ChatGPT-4o, particularly in relation to clinical functions, patient-facing applications, and perceived risks.

**Table 3:** Group comparisons between psychiatrists and psychologists.

Scale	Subscale	Subsection	Group	Mean ± SD	Test	Value	Df	Z	P	Effect size (d/r)
Profession			Psychiatrists	3.67 ± 0.58	t-test	-2.922	164		0.004	0.46
			Psychologists	3.40 ± 0.58						
As a Clinician-Facing Tool	Treatment	Psychoeducation	Psychiatrists	3.47 ± 0.95	t-test	-2.728	164		0.007	0.43
			Psychologists	3.01 ± 1.17						
As a Clinician-Facing Tool	Treatment	Structured Psychotherapeutic Support	Psychiatrists	3.78 ± 0.73	Mann–Whitney U	3173.5		0.623	0.534	
			Psychologists	3.62 ± 0.89						
As a Patient-Facing Tool			Psychiatrists	3.41 ± 0.58	t-test	-4.433	164	<0.001		0.68
			Psychologists	2.97 ± 0.70						
As a Patient-Facing Tool	Digital Access and Personalization in Youth Mental Health		Psychiatrists	3.55 ± 0.72	t-test	-3.588	164		<0.001	0.55
			Psychologists	3.12 ± 0.83						
Acting as a Therapist	Self-Help and Behavior Change Interventions		Psychiatrists	3.75 ± 0.74	Mann–Whitney U	2649.5		2.409	0.016	0.19
			Psychologists	3.47 ± 0.88						
Acting as a Therapist	Crisis Prevention and Safety Planning		Psychiatrists	3.54 ± 1.01	t-test	-4.471	164		<0.001	0.69
			Psychologists	2.75 ± 1.24						
Bias			Psychiatrists	2.29 ± 0.93	t-test	9.737	164		<0.001	1.56
			Psychologists	3.75 ± 0.93						

Independent samples t-tests were used where parametric assumptions were met and subscales were structurally equivalent across groups; Mann–Whitney U was applied otherwise. *p <.05. Effect sizes are reported as Cohen’s d for independent-samples t-tests and as r for Mann–Whitney U tests.

Child and adolescent psychiatrists reported a mean willingness to use ChatGPT-4o of 6.63, compared to 5.94 for psychologists; suggesting cautious openness (18). As shown in **Table 1**, child and adolescent psychiatrists strongly prioritized development in Software – diagnostic&treatment support (M = 8.93), system oversight (M = 8.82), and ethics (M = 8.51). Psychologists emphasized ethics (M = 8.50) and system oversight (M = 8.40), but were less enthusiastic about Software – Psychotherapy Support (M = 7.81). Consistent with Likert-based interpretation practices (15), values above 8 have been adopted in applied settings to indicate strong prioritization.

## 4 Discussion

This study extends existing ChatGPT-in-mental health discourse by integrating firsthand insights from Turkish professionals working in child and adolescent mental health. Diverging from earlier theory-driven approaches (13, 19, 20), our results offer grounded perspectives from practicing child and adolescent psychiatrists and psychologists, a significant portion of whom have incorporated ChatGPT-4o into their clinical routines. Consistent with prior findings (3), child and adolescent psychiatrists showed comparatively higher scores on the clinician-facing dimension, professional adaptation, and the potential of ChatGPT-4o to serve as a standalone psychotherapeutic agent, reflecting more favorable views in these domains. Within the psychologists, optimism was most pronounced in the bias and professional adaptation subscales relative to their responses on other domains. These outcomes align with broader patterns, underlining ChatGPT-4o’s promise in supporting psychiatric carexwhile reaffirming the necessity for supervised deployment (1, 21, 22). The comparative analyses further demonstrated that child and adolescent psychiatrists reported more favorable evaluations across several domains. Specifically, they more strongly endorsed the professional adaptation and the psychoeducational component of treatment, rated patient-facing applications more positively both at the total subscale level and within the Digital Access and Personalization in Youth Mental Health subsection, and placed greater emphasis on therapeutic applications such as self-help and behavior change interventions as well as crisis prevention and safety planning. In contrast, psychologists expressed comparatively higher optimism only within the bias subscale, while no notable group differences emerged for structured psychotherapeutic support. These discipline-specific patterns underscore how professional orientation may shape the perceived utility and risks of ChatGPT-4o in child and adolescent mental health practice (6, 14). These insights contribute a foundational step toward empirically rich research into the ethical, and professional challenges of adopting ChatGPT-4o in mental health care.

Clinician-facing tools evoke both interest and caution in mental health for their potential to streamline clinical workflows. In our study, child and adolescent psychiatrists strongly endorsed ChatGPT-4o’s diagnostic support, especially in differential diagnosis and organizing complex cases (1, 6), reflecting confidence in its role as a reasoning and synthesis tool. In contrast, psychologists showed lower agreement, especially on items C11 and C12, expressing concerns about diagnostic overreach and potential mislabeling (4, 23), reflecting a preference in the literature for interpretive depth over procedural speed (24, 25). While child and adolescent psychiatrists valued features like documentation and case formulation (M = 3.70), psychologists were more reserved, questioning the adequacy of LLMs for narrative-centered clinical tasks (6, 26). Consistent with prior sections, child and adolescent psychiatrists rated ChatGPT-4o -assisted treatment with moderately positive views (M = 3.79), driven in part by their greater recognition of medication guidance tools (M = 3.82), which align with their clinical responsibilities in pharmacological decision-making. Notably, this optimism extended to the psychoeducational component of treatment, where child and adolescent psychiatrists expressed comparatively higher endorsement than psychologists, suggesting that perceptions of value may be partly shaped by differences in clinical role and scope of practice. Psychologists, lacking prescriptive authority, rated this domain more neutrally (M = 3.16), indicating that perceptions of usefulness are influenced by professional scope. Still, both groups moderately supported the use of ChatGPT-4o as an assistive tool in psychotherapeutic contexts, reflecting shared acknowledgment of ChatGPT-4o’s utility in organizing therapy content (6, 14). Collectively, the findings underscore ChatGPT-4o’s growing relevance in structured psychiatric care and treatment planning, as an assistant (3, 20).

While ChatGPT-4o's clinician-facing applications are generally well received, its role as a patient-facing therapeutic agent elicited mixed views. Child and adolescent psychiatrists rated it moderately positively (M = 3.69), whereas psychologists remained neutral (M = 3.20). Beyond these mean differences, statistical comparisons demonstrated that child and adolescent psychiatrists scored significantly higher than psychologists on this dimension, with an effect size of *d* = 0.68, which corresponds to a moderate effect. Both groups showed ambivalence toward its capacity to build therapeutic alliance, underscoring uncertainty about its relational depth. Nonetheless, self-help behavioral interventions received moderate positive view, reflecting openness to low-intensity applications. This is consistent with literature praising AI’s accessibility but noting limitations in handling the emotional and ethical complexity of psychotherapy (27). Supporting this, evidence from anxiety-focused interventions reveals; while users report gains in cognitive restructuring, some also develop misplaced emotional trust, which may inadvertently delay professional engagement (13). Such dynamics echo ongoing academic concerns about AI’s inadequacy in delivering nuanced therapeutic responses which remains critical in high-quality mental health treatment (6). Extending these observations, child and adolescent psychiatrists also expressed moderately positive evaluations of crisis prevention and safety planning (M = 3.54), whereas psychologists’ ratings remained closer to neutrality (M = 2.75). This difference was statistically significant and corresponded to a moderate effect size (d = 0.69). This pattern resonates with prior work underscoring clinicians’ reliance on structured and protocol-driven decision support instruments, particularly in the management of risk and safety (6, 14, 20). Taken together, these findings illustrate discipline-specific orientations toward ChatGPT-4o, with child and adolescent psychiatrists displaying greater openness to its potential application in structured approaches to risk management.

Although both groups engaged with ChatGPT-4o, child and adolescent psychiatrists and psychologists showed clear contrast in their assessments of bias; child and adolescent psychiatrists reported a negative view (M = 2.29), while psychologists offered more favorable evaluations (M = 3.75). This difference represented the largest effect size observed across all comparisons (d = 1.56), underscoring the pronounced divergence between the two professions. One possible explanation for the magnitude of this effect lies in the fact that the bias subscale consisted of a single item, which may have inflated between-group differences due to limited variance capture and reduced measurement stability (28). Beyond measurement considerations, the disparity may also reflect differences in professional training and scope of practice. Child and adolescent psychiatrists, whose education emphasizes diagnostic reasoning and structured decision-making in high-stakes contexts, may be more attuned to risks of algorithmic distortion in sensitive clinical judgments. In contrast, psychologists, with training oriented toward therapeutic processes and interpretive depth, may view bias through a broader relational and contextual lens, which could foster relatively greater optimism regarding its manageability (6, 14, 20). Exposure to AI tools further differentiates the groups: child and adolescent psychiatrists more frequently encounter decision-support applications in pharmacological and acute care settings, whereas psychologists remain comparatively less exposed. This difference in exposure may shape their divergent appraisals of algorithmic fairness, amid concerns that ChatGPT-4o could amplify social bias through opaque data structures (3, 4). While some work suggests consistent prompts can mitigate bias (29), the absence of human benchmarks limits generalizability.

While enthusiasm for ChatGPT-4o expands, ethical scrutiny remains a key concern. In our findings, the ethical issues subscale scored lowest among both professional groups, emphasizing ongoing distrust regarding data safety and privacy (1). This supports existing literature warning of risks tied to transparency gaps, misinformation, and privacy vulnerabilities (3, 30). Furthermore, the absence of clear institutional protocols and regulatory frameworks in clinical settings likely contributes to professionals’ cautious stance, echoing prior reports that highlight gaps in institutional preparedness for AI adoption in healthcare (14, 20). Together, these results align with existing literature warning of risks tied to transparency gaps, misinformation, and privacy vulnerabilities, and they stress the urgent need for robust ethical guidelines and infrastructure before integration into routine mental health practice.

Our findings reveal a strong professional consensus favoring ChatGPT-4o as a supportive, not substitutive, tool in child and adolescent psychiatry. Child and adolescent psychiatrists prioritized diagnostic&treatment software, ethics, and system oversight (19), while psychologists shared ethical concerns and system oversight. These insights highlight the value of profession-specific ChatGPT-4o development. Still, the moderately positive stance on professionalism, alongside neutral evaluations in general impression and willingness, suggests both groups see potential but remain cautious about clinical adoption. This measured outlook reflects earlier findings indicating that openness to AI often coexists with hesitation (14, 31).

As with many early investigations, this study has certain methodological constraints. These results capture clinician perspectives at a single point in time, limiting the understanding of how views may evolve as ChatGPT-4o progresses. Additionally, the absence of behavioral metrics restricts insights into real-world clinical applicability. Recruitment via purposive and convenience sampling through WhatsApp groups may also introduce selection bias, favoring digitally engaged participants. Furthermore, thematic analysis was not conducted due to the limited sample size, which constrained the ability to derive reliable codes. Similarly, content and construct validity procedures, such as factor analysis, were not performed due to limited item numbers per subscale and sample size, which may compromise statistical stability in exploratory settings (32). While, high internal consistency in the scales (Cronbach’s alpha > 0.80) supports the robustness of the findings (33), three subscales demonstrated lower internal consistency (α = 0.491, α = 0.512, and α = 0.577), which aligns with findings in early-stage exploratory instruments with fewer items per subscale (32). Yet, within the scope of exploratory research, timely and well-structured clinician input offers valuable preliminary direction. Notably, conducted approximately one year after ChatGPT-4o’s debut, this study stands among the earliest empirical investigations into its perceived value in child and adolescent mental health.

## 5 Conclusions

In summary, this research offers a grounded, practice-informed perspective on how child and adolescent mental health professionals perceive the clinical incorporation of ChatGPT-4o. Child and adolescent psychiatrists stressed its value in clinician-oriented and therapeutic contexts, while psychologists’ higher bias ratings may signal trust in its objectivity. Although ethical and boundary-related disadvantages persist, clinicians—especially psychiatrists—expressed cautious optimism regarding its use in diagnostic support and treatment planning. The results provide actionable direction for responsibly incorporating ChatGPT-4o into child and adolescent mental health care, underscoring its potential as an assistive tool. To translate these insights into practice, the development of structured training initiatives tailored to different professional roles may enhance the effective integration of ChatGPT-4o (6, 14). Beyond individual competencies, profession-specific guidelines and institutional protocols are required to promote safe, ethically grounded, and context-sensitive adoption in clinical care. Successful use will require context-aware protocols, and well-defined practice boundaries (20). Given that this study relied on a non-probability sample within a country-specific context, the findings should be interpreted as exploratory and not assumed to generalize to global practice. Future investigations should not only adopt longitudinal approaches to monitor how professional perspectives evolve as ChatGPT-4o becomes integrated into everyday clinical workflows, but also initiate pilot implementation studies within real-world practice. Such initiatives would provide essential evidence on feasibility, safety, and therapeutic impact, thereby informing context-sensitive guidelines and supporting policy development for responsible integration.

At the local level, this study contributes rare empirical evidence from Turkish clinicians, offering insights into an underexplored professional group. At the national level, the findings provide timely input that can guide AI-related discussions on training, regulation, and clinical governance in Turkey’s mental health system. At the global level, the study adds a non-Western, practice-based perspective to the emerging literature on AI in child and adolescent mental health, thereby expanding the diversity and generalizability of knowledge in this domain.

## Data Availability

The raw data supporting the conclusions of this article will be made available by the authors, without undue reservation.
