# Correction: From promise to practice: insights into ChatGPT-4o use in child and adolescent mental health from professionals

**DOI:** 10.3389/fpsyt.2025.1717346

**Published:** 2025-11-27

**Authors:** Armagan Aral, Gizem Gerdan, Mirac Barıs Usta, Ayse Erguner Aral

**Affiliations:** 1 Department of Child and Adolescent Psychiatry, City Hospital of Izmır, Izmır, Türkiye; 2 Department of Psychology, Division of Clinical Psychology, Izmır Democracy University, Izmır, Türkiye; 3 Department of Child and Adolescent Psychiatry, Ondokuz Mayıs University, Faculty of Medicine, Samsun, Türkiye; 4 Department of Psychiatry, City Hospital of Izmır, Izmır, Türkiye

**Keywords:** ChatGPT-4o, child and adolescent, clinical integration, mental health, professional perspectives

There was a mistake in the figures as published. The article was missing the **Graphical Abstract**. The **Graphical Abstract** appears below.

**Graphical Abstract:**
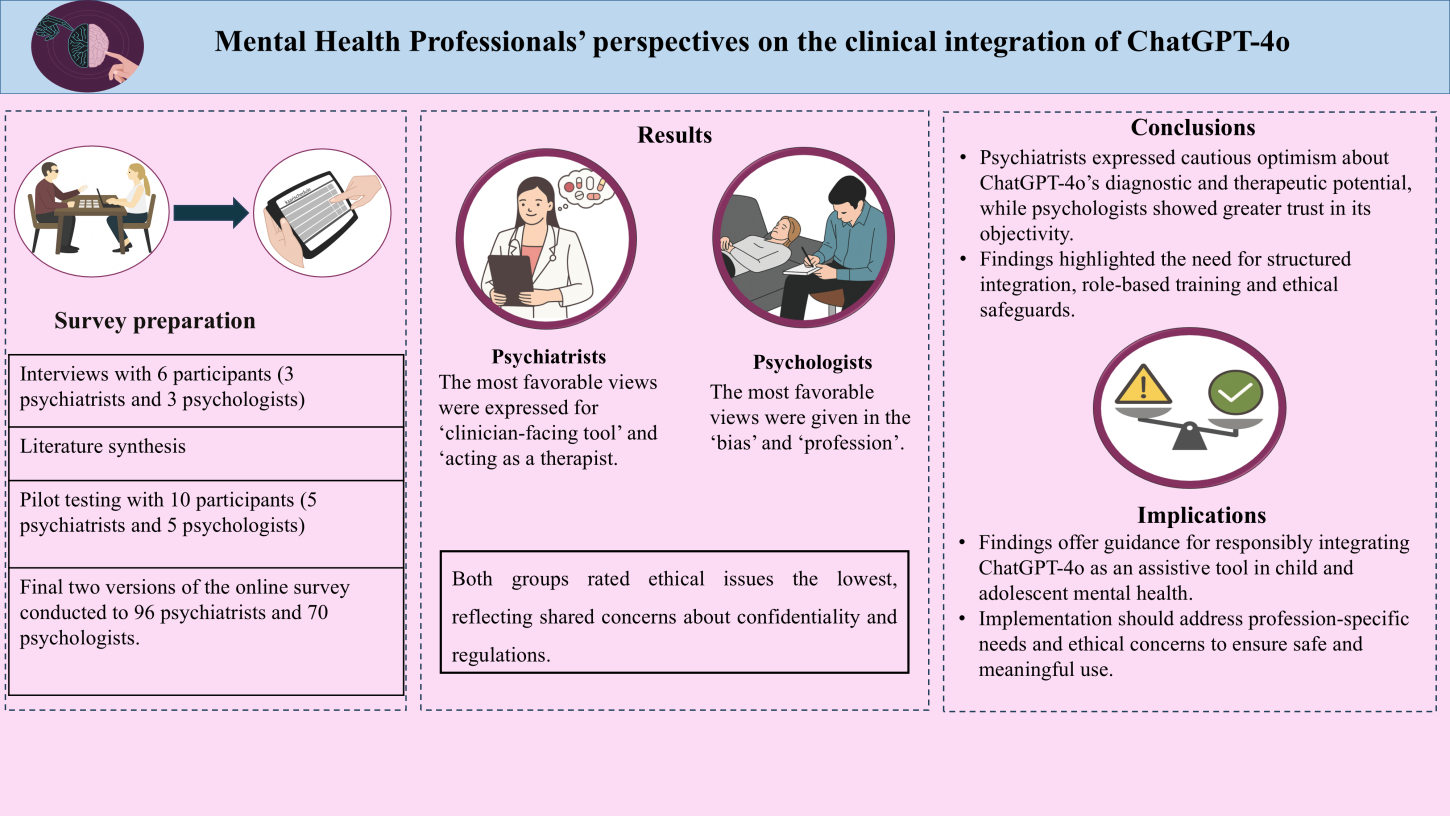


There was a mistake in **Figure 1** as published. There was a spelling error in the title of the donut chart. “Opnions on ChatGPT-40 Use in Clinical Practice” should be “Opinions on ChatCGPT-4o in Clinical Practice”. The corrected **Figure 1** appears below.

**Figure 1:**
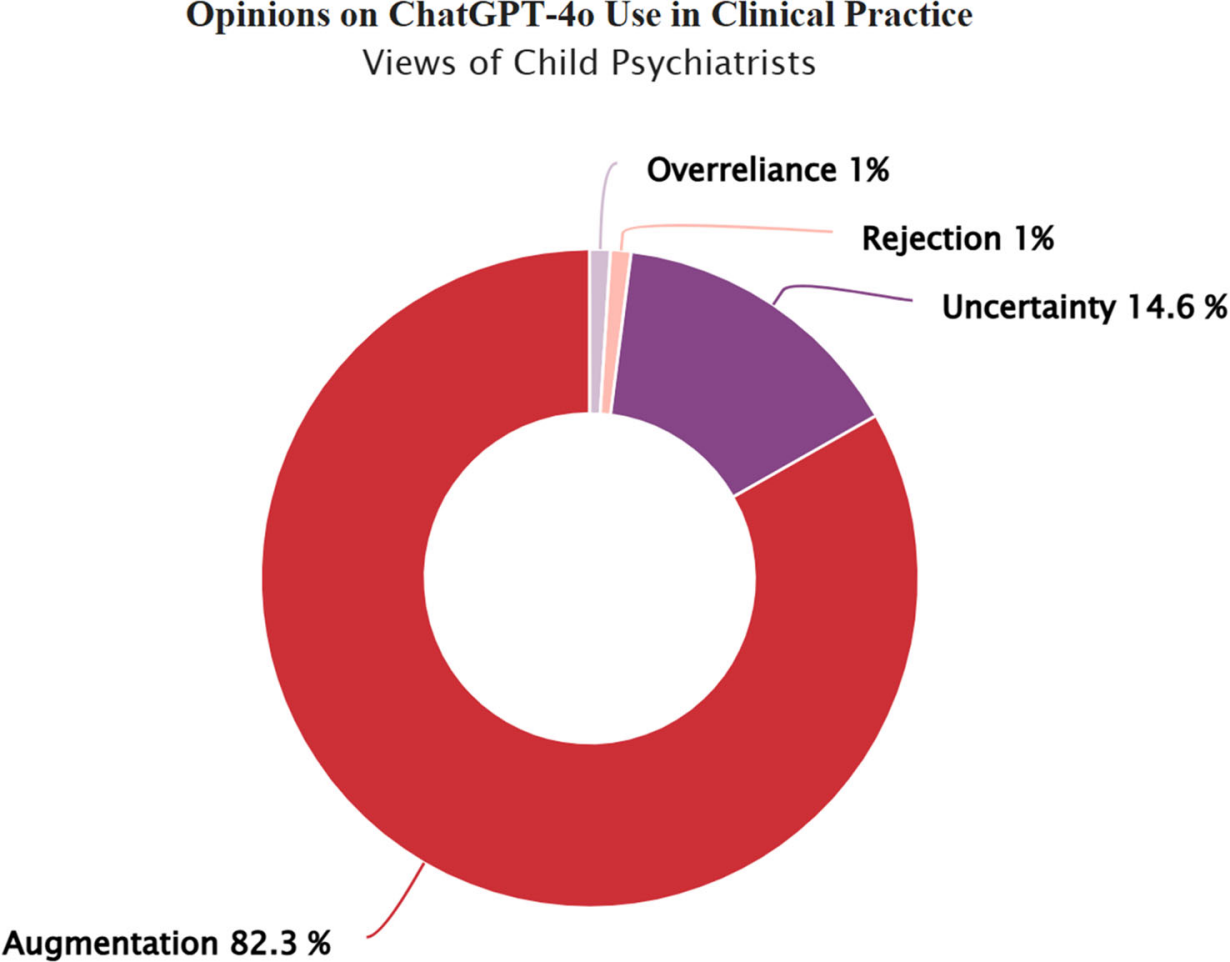
Child psychiatrists’ views on integrating ChatGPT-4o into clinical practice. Child psychiatrists’ views on integrating ChatGPT-4o into clinical practice. Response categories reflect varying perspectives on the integration of ChatGPT-4o in child and adolescent mental health practice: Rejection: ChatGPT-4o has no place in clinical practice, Overreliance: Unquestioning trust in ChatGPT-4o’s diagnostic and treatment suggestions, Uncertainty: Uncertainty regarding its clinical usefulness, Augmentation: A synergistic effect could emerge by combining mental health professionals’ clinical expertise with ChatGPT-4o’s analytical capabilities.

There was a mistake in **Figure 2** as published. **Figure 2** should have been the graphical abstract. The correct **Figure 2** appears below.

**Figure 2:**
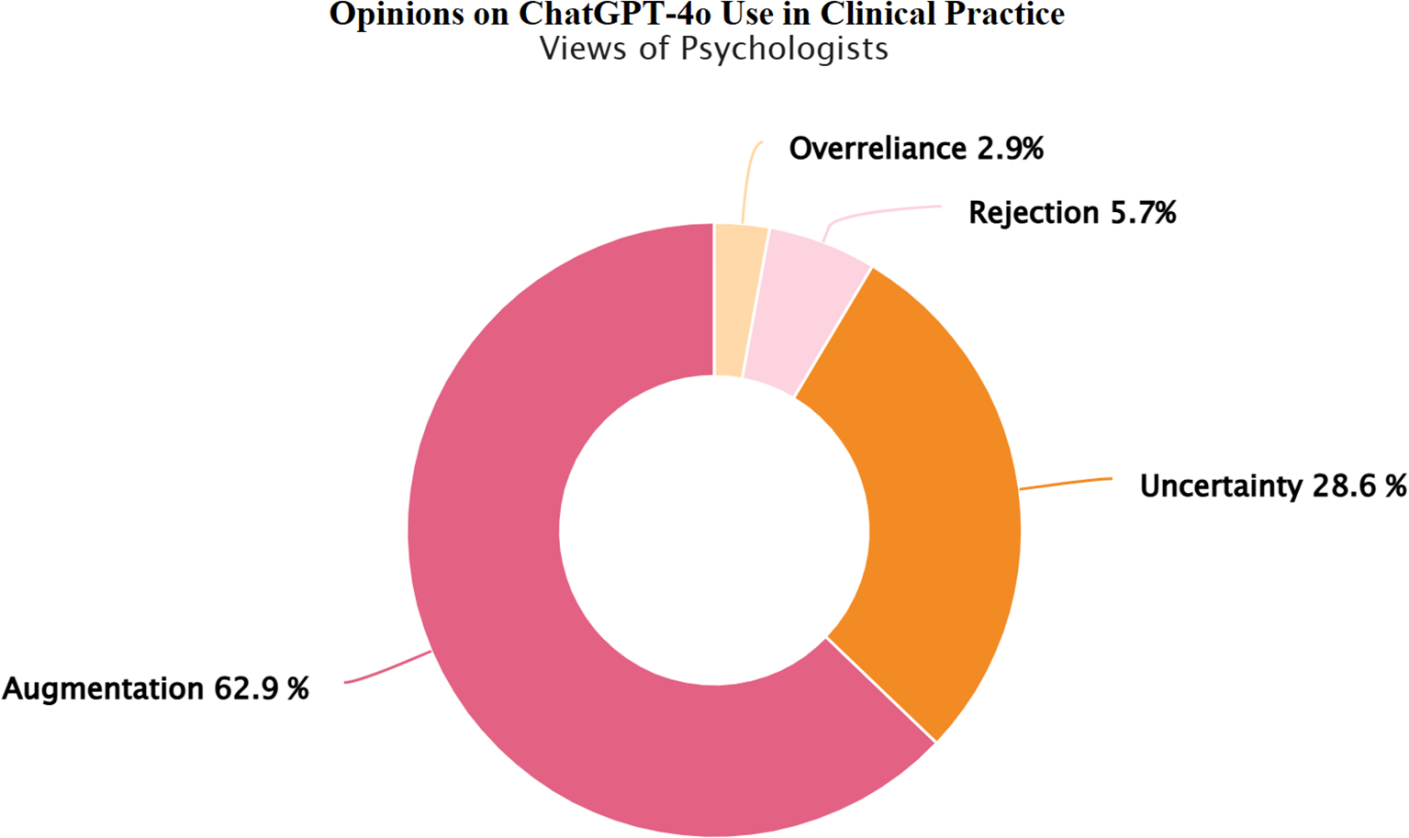
Psychologists’ views on integrating ChatGPT-4o into clinical practice. Psychologists’ views on integrating ChatGPT-4o into clinical practice. Response categories reflect varying perspectives on the integration of ChatGPT-4o in child and adolescent mental health practice: Rejection: ChatGPT-4o has no place in clinical practice, Overreliance: Unquestioning trust in ChatGPT-4o’s diagnostic and treatment suggestions, Uncertainty: Uncertainty regarding its clinical usefulness, Augmentation: A synergistic effect could emerge by combining mental health professionals' clinical expertise with ChatGPT-4o’s analytical capabilities.

The original version of this article has been updated.

