# Supplementary material for: From promise to practice: insights into ChatGPT-4o use in child and adolescent mental health from professionals
Source: Front Psychiatry. 2025 Sep 26;16:1668814. doi: 10.3389/fpsyt.2025.1668814 (PMC12511095; doi:10.3389/fpsyt.2025.1668814)
Supplement: Supplementary file 1 [file DataSheet1.docx]

Supplementary Material-1

**Table S1. Interviewee Characteristics**

| Nickname | Gender | Age(years) | Professional experience (years) | Professional title | Affiliated Institution | Interest in ChatGPT-4o | Interview Duration (minute) |
| --- | --- | --- | --- | --- | --- | --- | --- |
| PSY-1 | Female | 38 | 13 | Associate Professor | State Hospital | Academic writing | 40 |
| PSY-2 | Male | 42 | 5 | Specialist doctor | State Hospital | Administrative tasks | 55 |
| PSY-3 | Male | 30 | 5 | Specialist doctor | State Hospital | Administrative tasks | 50 |
| PSL-1 | Female | 38 | 11 | Clinical Psychologist | Private Practice | Clinical practice | 43 |
| PSL-2 | Female | 41 | 19 | Assistant Professor | Private Practice | Clinical practice- Preparing presentations | 45 |
| PSL-3 | Female | 32 | 9 | Clinical Psychologist | Private Practice | Academic writing | 43 |

Note. PSY1–3 for psychiatrists, PSL1–3 for psychologists

**Verbatims**

**From Promise to Practice: Insights into ChatGPT-4o Use in Child and Adolescent Mental Health from Professionals**

This study aims to explore the views of professionals working in the field of child and adolescent mental health regarding the integration of ChatGPT-4o, an artificial intelligence-based chatbot, into clinical practice. The questions presented in this research are designed to reveal your professional experiences, insights, and ethical evaluations. You are kindly asked to share your opinions about the draft survey before its finalization and to suggest any additional questions, if applicable. The collected data will be used to develop a comprehensive framework regarding the potential role of AI-supported systems in child and adolescent mental health care. Participation is entirely voluntary, and your responses will be used solely for scientific purposes.

**Participant:PSY-1**

**Name-Surname**: G**O**-PSY-1
**Age**: 38
**Gender**: Female
**How many years have you been working in child and adolescent mental health**: 13
**Title**: Resident Doctor / Research Assistant / Specialist Doctor / Assistant Professor / Associate Professor / Professor
**Institution**: Private Practice / State Hospital / University Hospital

**Have you used ChatGPT before**: Yes
**If yes, for what purpose?**: In clinical practice / For writing academic articles / For preparing presentations / For administrative tasks (e.g., tables, annual planning, etc.)

**1. What are your general views on the integration of ChatGPT-4o (an AI-based chatbot) into clinical practice as a child and adolescent mental health professional? What role do you think these tools could play in your profession?**

The use of ChatGPT-4o, an AI-supported chatbot, in the field of child and adolescent mental health represents a novel development that must be carefully evaluated. Such systems could contribute significantly to diagnosis and treatment by analyzing electronic health records, scientific resources, and up-to-date treatment guidelines. Especially in terms of accelerating access to information, I believe these tools will play a more active role as supportive instruments in future clinical practice. Their potential to integrate evidence-based practices and current methodologies into such systems is also valuable. However, concerns that these technologies may limit clinicians’ analytical thinking and creative problem-solving abilities must not be overlooked. Despite all the risks, I believe that these tools may provide significant benefits to both professionals and clients. When human judgment remains central to decision-making processes, the ethical and conscious integration of AI can meaningfully support professional practice.

**2. What do you see as the advantages and potential risks of using ChatGPT-4o in diagnosis, treatment planning, and patient management in child and adolescent mental health?**

While ChatGPT cannot directly make diagnoses, it may assist in the process by accelerating diagnostic assessments and generating more targeted questions. Its ability to detect emotional tones in text and explain those insights could be useful in clinical evaluations. Additionally, it can guide clinicians by offering treatment options based on current guidelines, dosage information, and indications. In structured, skill-building approaches like CBT, it can be effective in delivering psychoeducational and practical content. Its personalized suggestions for managing anger and anxiety can also strengthen client support.
However, generalized recommendations that fail to sufficiently account for individual differences represent a major limitation. The inability to apply creative therapeutic techniques—such as metaphors or storytelling—may hinder the process. Moreover, there is a risk of pathologizing normative experiences. Some clients may feel negatively about confiding in a chatbot rather than a human therapist.
Nonetheless, ChatGPT’s capacity to offer consistent and empathetic responses, independent of the therapist’s emotional state, fatigue, or biases, may prove valuable in specific clinical scenarios. Its potential contribution to safety planning for clients at risk of self-harm is also a significant advantage.

**3. What are your thoughts on the direct use of ChatGPT-4o by child and adolescent clients or their families? Do you think these tools can be helpful for guidance, information, and support, or might they have negative effects on the psychiatric care process?**

The direct use of ChatGPT-4o by children, adolescents, and their families presents both advantages and important risks. Its availability at any time and patient handling of repetitive questions can be especially helpful for time management. Allowing clients and families to gain preliminary information before consulting a professional may help them approach the process more prepared. Access to timely, understandable, and pressure-free information can empower individuals to make informed mental health decisions. Children and adolescents growing up in the digital age are more naturally inclined toward such technologies when seeking psychological support.
However, responses shaped by user input—even if the system can correct errors—may lead vulnerable individuals into unnecessary or illogical arguments, presenting psychiatric risks. Excessive reliance on such tools may delay or prevent seeking professional help when needed. Therefore, the boundaries of using systems like ChatGPT for information and support must be clearly defined. It should be clearly stated that professional evaluation and guidance are required when clinical concerns arise.

**4. Do you think ChatGPT-4o could be effective for clinical documentation and medical record management? What are the main challenges in practical use?**

Although ChatGPT-4o may offer advantages for clinical documentation and record-keeping, several practical and ethical concerns accompany its use. Its ability to help clinicians generate case formulations more systematically and efficiently can reduce workload. However, transferring client information into such a system may be impractical and raises serious confidentiality concerns. For professionals working in child and adolescent mental health, client privacy is critical, and families may feel uneasy about their personal data being processed in digital environments.
Considering current ethical standards and professional norms, the active use of ChatGPT in these processes may be deemed unethical. Therefore, any application of such technologies in clinical documentation must strictly uphold patient rights and ethical responsibilities.

**5. What regulations, training, or technical improvements are necessary for more effective future use of AI-based systems in child and adolescent mental health? What aspects of ChatGPT-4o should be improved to better support your professional work?**

To enhance the effective use of AI in psychiatry, both technical advancements and ethical regulations are needed. AI systems must be equipped with diagnostic and treatment planning algorithms to ensure clinical accuracy and reliability. Enhancing their role in psychoeducation would also help individuals gain knowledge and develop skills related to mental health.
Professional training programs should be developed to improve mental health professionals' competency in using AI tools. Additionally, the use of AI in psychiatry raises complex ethical issues that must be addressed not only technically, but also legally and professionally. An interdisciplinary approach to resolving ethical concerns will support the responsible integration of these systems into clinical practice.

**6. Do you have any additional questions you think should be included beyond those already asked?**

If ChatGPT were to be used for providing mental health support, how do you envision this impacting the role of mental health professionals?

**Interview Duration**: 40 minutes
**Date**: 28.03.2025

**Participant:PSY-2**

**Name-Surname**: H**M**A**-PSY-2
**Age**: 42
**Gender**: Male

**How many years have you been working in child and adolescent mental health**: 5
**Title**: Resident Doctor / Research Assistant / Specialist Doctor / Assistant Professor / Associate Professor / Professor
**Institution**: Private Practice / State Hospital / University Hospital

**Have you used ChatGPT before**: Yes
**If yes, for what purpose?**: In clinical practice / For writing academic articles / For preparing presentations / For administrative tasks (e.g., tables, annual planning, etc.)

**1. What are your general views on the integration of ChatGPT-4o (an AI-based chatbot) into clinical practice as a child and adolescent mental health professional? What role do you think these tools could play in your profession?**

ChatGPT-4o should not replace a qualified specialist in the field of child and adolescent mental health; however, it can be integrated into the clinical process as a supportive tool. When used appropriately, it may enhance professional decision-making and improve accessibility. Nonetheless, delegating patient assessment to artificial intelligence is not considered safe. Within the framework of ethical guidelines, its limited and controlled use represents the most appropriate approach.

**2. What do you see as the advantages and potential risks of using ChatGPT-4o in diagnosis, treatment planning, and patient management in child and adolescent mental health?**

ChatGPT-4o may be beneficial in generating differential diagnosis lists, suggesting appropriate medication options, and providing personalized recommendations. However, its inability to detect certain mental disorders may lead to missed diagnoses. Moreover, its lack of sensitivity to nonverbal cues could weaken the patient-clinician relationship. In cases of uncontrolled use, inaccurate suggestions may pose psychological risks.

**3. What are your thoughts on the direct use of ChatGPT-4o by child and adolescent clients or their families? Do you think these tools can be helpful for guidance, information, and support, or might they have negative effects on the psychiatric care process?**

ChatGPT-4o can facilitate families' access to information about their children's mental health and help them come to appointments better prepared. Its ability to provide evidence-based recommendations makes it a potentially informative tool. However, the presence of biased or inaccurate content poses a risk of misinformation. Therefore, its direct use without professional supervision should be approached with caution.

**4. Do you think ChatGPT-4o could be effective for clinical documentation and medical record management? What are the main challenges in practical use?**

ChatGPT-4o may streamline the documentation process by accelerating case formulations. However, it may not always accurately capture clinically meaningful details, potentially compromising the integrity of the patient’s narrative. Furthermore, to ensure ethical use, the software must be further developed and subjected to professional oversight.

**5. What regulations, training, or technical improvements are necessary for more effective future use of AI-based systems in child and adolescent mental health? What aspects of ChatGPT-4o should be improved to better support your professional work?**

To enhance the future effectiveness of ChatGPT-4o, it is essential that the system be trained specifically for psychotherapy processes. Software improvements should be implemented to strengthen its documentation capabilities and ensure content accuracy. Professional oversight and regular monitoring are crucial to ensure safe use. In this way, the tool can support clinical practice while maintaining ethical boundaries.

**6. Do you have any additional questions you think should be included beyond those already asked?**

No

**Interview Duration**: 55 minutes
**Date**: 24.03.2025

**Participant:PSY-3**

**Name-Surname**: H**I**C**-PSY-3
**Age**: 30
**Gender**: Male

**How many years have you been working in child and adolescent mental health**: 5
**Title**: Resident Doctor / Research Assistant / Specialist Doctor / Assistant Professor / Associate Professor / Professor
**Institution**: Private Practice / State Hospital / University Hospital

**Have you used ChatGPT before?**: Yes
**If yes, for what purpose?**: In clinical practice / For writing academic articles / For preparing presentations / For administrative tasks (e.g., tables, annual planning, etc.)

**1. What are your general views on the integration of ChatGPT-4o (an AI-based chatbot) into clinical practice as a child and adolescent mental health professional? What role do you think these tools could play in your profession?**

The integration of ChatGPT-4o into child and adolescent psychiatry practice must be approached with caution and responsibility. AI-based tools such as this offer significant opportunities to enhance access to mental health services and improve the quality of care provided. When appropriately integrated, they may also contribute to reducing clinical errors within treatment processes. However, it is by no means appropriate for artificial intelligence to replace professional mental health services. Delegating highly sensitive tasks—such as patient assessment—entirely to such systems may pose serious risks. Nonetheless, these technologies carry considerable potential to enrich and support professional practice. Therefore, mental health professionals must remain open to such innovations and be willing to engage with these tools in a conscious and informed manner to keep pace with digital advancements in psychiatry.

**2. What do you see as the advantages and potential risks of using ChatGPT-4o in diagnosis, treatment planning, and patient management in child and adolescent mental health?**

The use of ChatGPT-4o in diagnosis, treatment planning, and patient management within child and adolescent mental health presents clear advantages alongside several critical risks. Its ability to generate comprehensive and accurate differential diagnoses, particularly in complex cases, stands out as a supportive feature in clinical evaluation. Additionally, its capacity to guide appropriate medication selection and provide detailed information on side effects, potential adverse reactions, changes in laboratory findings, or drug interactions renders it a valuable resource for clinicians.
On the other hand, its potential failure to accurately identify certain psychiatric disorders may result in missed diagnoses and negatively affect treatment planning. Moreover, as the system is unable to interpret nonverbal cues that play a crucial role in face-to-face encounters, it may weaken the therapeutic alliance and disrupt the collaboration between patient and clinician.
That said, while medical professionals sometimes communicate using overly technical or complex terminology—making information less accessible for patients—ChatGPT can simplify and clarify such content, facilitating understanding. Its immunity to personal factors such as fatigue, mood, or subjective experience may also allow it to generate more consistent and empathetic responses in certain contexts.
Furthermore, its capacity to offer personalized suggestions regarding behavioral activation or sleep hygiene is notable. Nevertheless, unsupervised use of the system carries the risk of delivering inaccurate or inappropriate recommendations, which could heighten anxiety or even trigger self-harming behaviors in vulnerable individuals.

**3. What are your thoughts on the direct use of ChatGPT-4o by child and adolescent clients or their families? Do you think these tools can be helpful for guidance, information, and support, or might they have negative effects on the psychiatric care process?**

The direct use of ChatGPT-4o by child and adolescent patients and their families can offer meaningful contributions to information dissemination and support, as long as it remains within appropriate boundaries. It may serve as a valuable resource, particularly for parents and caregivers, by helping them better understand their child’s mental health processes and access guidance as they navigate these challenges. Furthermore, such conversation-based systems can help patients and families arrive at psychiatric consultations more informed and prepared, thereby enhancing the quality of communication with clinicians.
While ChatGPT has the capacity to provide individualized, evidence-based treatment suggestions, it is essential to acknowledge that diagnostic errors—though sometimes inevitable—must be recognized and evaluated in comparison with professional “gold standard” assessments. Additionally, given that AI models are trained on existing digital content, they may unintentionally absorb and reflect stigmatizing narratives, discriminatory language, or misinformation regarding mental illness, which could reinforce harmful biases in online environments.
For this reason, ChatGPT should not be seen as a replacement for professional mental health care. Rather, it should be considered a carefully supervised and guideline-oriented tool for support and education.

**4. Do you think ChatGPT-4o could be effective for clinical documentation and medical record management? What are the main challenges in practical use?**

ChatGPT-4o offers certain advantages in clinical record-keeping and documentation processes. In particular, its ability to generate case formulations in a more practical and structured manner can be time-saving for mental health professionals. However, it is important to recognize that such AI systems may not fully capture the unique and clinically significant details embedded in a patient’s narrative. This limitation may hinder a comprehensive understanding of the case’s psychosocial context and compromise a holistic assessment. Therefore, clear ethical boundaries must be established for the use of AI in treatment and documentation, and explicit regulations should be developed to govern its application. Only then can the safe and responsible integration of such technologies into clinical practice be ensured.

**5. What regulations, training, or technical improvements are necessary for more effective future use of AI-based systems in child and adolescent mental health? What aspects of ChatGPT-4o should be improved to better support your professional work?**

To enable more effective use of AI-based systems in the field of child and adolescent mental health, it is essential to strengthen the technical infrastructure and ensure professional oversight. Enhancing content related to psychotherapy with higher-quality data will facilitate the supportive use of these technologies in therapeutic work. Similarly, software improvements in clinical documentation will contribute to more efficient and reliable record-keeping processes. However, for these systems to function effectively, their content must be reviewed by qualified professionals, and the AI applications must be subject to continuous monitoring. Without such oversight and regular updates, it will not be possible for artificial intelligence to serve as a safe and sustainable tool in mental health services.

**6. Do you have any additional questions you think should be included beyond those already asked?**

In your opinion, what role should ChatGPT-4o assume, or what position should it occupy, within clinical practice in the field of child and adolescent psychiatry?

**Interview Duration**: 50 minutes
**Date**: 24.03.2025

**Participant:PSL-1**

**Name-Surname:** A**K**

**Age:** 38

**Gender:** Female

**How many years have you been working in child and adolescent mental health:** 11

**Title:** Research Assistant / Psychologist /Clinical Psychologist / Assistant Professor / Associate Professor / Professor

**Institution**: Private Practice / State Hospital / University Hospital/Department of Psychology, University/ Ministry of Family and Social Services/School/ Rehabilitation Center

**Have you used ChatGPT before:** Yes

**If yes, for what purposes?** In clinical practice / For writing academic articles / For preparing presentations / For administrative tasks (e.g., tables, annual plans, etc.)

**1.What are your general views on the integration of ChatGPT-4o (an AI-based chatbot) into clinical practice as a child and adolescent mental health professional? What role do you think these tools could play in your profession?**

It is a program I rarely use in clinical practice, but I believe it facilitates quicker access to theoretical knowledge. Currently, I mostly use it for managing official processes in the center.

**2.What are the potential advantages and risks of using ChatGPT-4o in the processes of diagnosis, treatment planning, and patient management in child and adolescent mental health?**

It can be useful for reviewing diagnostic criteria and obtaining concrete information. It may also be used to prepare informational texts for families and to write content related to developmental characteristics of children and adolescents. It can facilitate time management.When it comes to treatment planning, I focus on clinical observations and understanding the emotional needs of the client. While working with children and adolescents, I also engage with the family system. In these settings, understanding their desires, fantasies, and needs, addressing their projections, and creating space for transformation is central to my work. I believe using ChatGPT could lead to confusion or superficial interpretations, focusing too much on what is apparent. It might limit the therapist’s personal associations and make it harder to reflect on and relate to the client. This could hinder a holistic approach. Perhaps that’s why I rarely use it in my clinical work. Care must also be taken regarding confidentiality and privacy**.**

**3.What do you think about the direct use of ChatGPT-4o by child and adolescent clients or their families? Do you believe this tool could be helpful in providing guidance, information, and support, or might it pose risks to the therapeutic process?**

It can offer both fast and comprehensive explanatory texts for guidance and information. I believe it can be facilitative. When clients struggle with self-reflection, it may help meet their immediate needs. It could serve as a step toward understanding their own mental health needs, raising awareness, and eventually seeking help when necessary.Conversely, it may also lead to delays in treatment due to reliance on quick behavioral solutions and recommendations.

**4.Do you think ChatGPT-4o could be effective in clinical documentation and medical record management? What are the main practical challenges that could arise from using such systems?**

It could definitely be practical in terms of usability. However, considering that confidentiality and privacy are among the most fundamental principles of our profession, transferring all data to such a platform might be risky. In the long run, it may affect the trust relationship between the therapist and the client. I would want to research the program to ensure protection for both the client and the practitioner.

**5.What regulations, training, or technical improvements are necessary to ensure more effective use of AI-based systems in child and adolescent mental health in the future? What aspects of ChatGPT-4o should be improved to better support your professional work?**

It would be great if it could provide references.

**6. Do you have any suggestions for additional questions that should be added to the current ones?**

None.

**Interview duration:** 43 minutes

**Date:** 23.03.2025

**Participant:PSL-2**

**Name-Surname:** B**E**

**Age:** 41

**Gender:** Female

**How many years have you been working in child and adolescent mental health:** 19

**Title:** Research Assistant / Psychologist /Clinical Psychologist / Assistant Professor / Associate Professor / Professor

**Institution**: Private Practice / State Hospital / University Hospital/Department of Psychology, University/ Ministry of Family and Social Services/School/ Rehabilitation Center

**Have you used ChatGPT before:** Yes

**If yes, for what purposes?** In clinical practice / For writing academic articles / For preparing presentations / For administrative tasks (e.g., tables, annual plans, etc.)

**1.What are your general views on the integration of ChatGPT-4o (an AI-based chatbot) into clinical practice as a child and adolescent mental health professional? What role do you think these tools could play in your profession?**

AI-based systems may play a significant role as potential support tools in the field of child and adolescent mental health. However, their effective integration requires careful attention to ethical, safety, and confidentiality concerns. Moreover, artificial intelligence can never replace human interaction; therefore, as professionals, the most appropriate approach is to view this technology as a complementary—not substitutive—resource.

- I believe that artificial intelligence can assist in early diagnosis processes by analyzing surveys or specific criteria to assess the mental states of children and adolescents.
- Its accessibility may allow more young individuals to benefit, which can increase access to mental health services—especially for children living in rural areas.
- Chatbots can engage in continuous interaction with individuals, recording current emotional states and facilitating expert monitoring. They may also be useful in emergencies by providing instant support.
- By informing users about mental health, they can raise awareness and provide education. Children and young people may become better informed about mental health issues.
- Experts can use data from artificial intelligence to monitor individuals’ emotional and behavioral development during the therapy process.

**Possible Challenges**

- It must be clarified how the privacy of children’s and adolescents’ personal data will be protected. How artificial intelligence processes and stores this data should be carefully addressed.
- Since the emotional support capabilities of artificial intelligence are limited, it cannot function like a mental health professional. Human relationships are highly important in mental health services; therefore, AI should only be used as a supportive tool.
- AI-based systems carry the risk of providing incorrect information or misinterpreting situations. Such issues must be carefully managed during the monitoring and evaluation process.

**2.What are the potential advantages and risks of using ChatGPT-4o in the processes of diagnosis, treatment planning, and patient management in child and adolescent mental health?**

- It can help mental health professionals obtain faster feedback and results during diagnostic processes.
- By analyzing risk factors and symptom clusters, it can support the early identification of problems in children and adolescents.
- Artificial intelligence can use individual data to develop personalized treatment and intervention plans. This can contribute to a more effective treatment process.
- It can continuously monitor and analyze patients’ mental states. This process may enable better patient follow-up through regularly updated data.

However, it may be difficult to ensure data security and prevent misuse within AI systems.
AI systems may not fully understand complex human behaviors and mental states. Misdiagnoses or incorrect suggestions can lead to negative outcomes for individuals.
AI cannot fully provide human-specific skills such as empathy and emotional understanding. This inevitably creates shortcomings in the therapeutic process.

**3.What do you think about the direct use of ChatGPT-4o by child and adolescent clients or their families? Do you believe this tool could be helpful in providing guidance, information, and support, or might it pose risks to the therapeutic process?**

While ChatGPT-4o and similar AI-based systems hold great potential for providing information and support to child and adolescent clients and their families, careful and conscious use is essential. These AI tools can be considered complementary to mental health services but should never replace professional help. Supporting users with accurate information and guidance on how to use these tools is critical in minimizing potential negative effects.

**Benefits:**

- Provides Information and Raises Awareness:

AI can offer families and clients information about mental health issues, treatment processes, and coping strategies. This can help them make informed decisions and better understand their problems.

- Accessibility:

Especially for families living in rural or remote areas, providing access to online resources and information may facilitate access to mental health services.

- Privacy and Anonymity:

Children and adolescents may feel more comfortable interacting with AI. Their willingness to speak openly about certain topics may increase, making them feel more at ease.

- Quick Response and Support:

In emergencies or moments of crisis, AI can provide immediate support to help users better manage their situations. For example, it can offer instant advice related to stress or anxiety.

- Self-Help Tools:

Users can learn various techniques to support themselves. Resources such as breathing and relaxation exercises, meditation techniques, stress management tips, or emotion journaling can help individuals manage their own mental health.

**Risks:**

- AI systems may provide incorrect information or fail to offer appropriate support regarding complex mental health problems. This can lead to misguidance or misunderstandings.
- AI cannot offer essential human skills such as empathy and emotional understanding.
- In cases of emergencies or serious mental health conditions, AI may be insufficient in providing the necessary support. It is crucial to emphasize that professional help should be sought in such situations.
- It may be difficult for families to monitor and manage their children’s interactions with AI systems. This could impact the parent-child relationship and reduce the effectiveness of family involvement in the mental health process.

**4.Do you think ChatGPT-4o could be effective in clinical documentation and medical record management? What are the main practical challenges that could arise from using such systems?**

AI-Based Systems Like ChatGPT-4o May Be Effective in Clinical Documentation and Medical Record Management

- AI systems can automate patient information and clinical data entry, saving time. This allows healthcare professionals to devote more time to direct patient care.
- Based on predefined rules and templates, AI can provide greater consistency and accuracy in the documentation process. This may contribute to the standardization of patient records.
- With advanced data analysis capabilities, AI can analyze clinical data to provide insights into trends, outcomes, and patient progress—useful for optimizing treatment plans.
- AI can enable fast search and retrieval of medical records, allowing healthcare professionals to quickly access the information they need.
- By generating automated reporting processes, AI can facilitate the preparation of statistics and outputs based on patient data at specific intervals more efficiently.

Key Challenges:

- Health data is highly sensitive and subject to privacy concerns. It is critical that AI systems have adequate security measures in place to protect patient information.
- AI systems may misinterpret information entered into medical records or introduce errors in data input, potentially endangering patient safety. Therefore, human oversight of the processes is essential.
- Healthcare professionals must receive adequate training to use AI tools effectively. A lack of training may lead to misuse or reduced effectiveness of these tools.
- The use of AI systems with patient data can raise ethical issues. Additionally, compliance with legal regulations governing the digitization and management of medical records is necessary.
- As AI usage increases, the human factor—namely, the direct involvement of healthcare professionals—may be overlooked. It is crucial that AI does not replace human interaction in patient care.

**5.What regulations, training, or technical improvements are necessary to ensure more effective use of AI-based systems in child and adolescent mental health in the future? What aspects of ChatGPT-4o should be improved to better support your professional work?**

Laws, principles, ethical guidelines, and standards should be established to ensure the privacy of personal data. Training programs should be provided for both professionals and users. Since artificial intelligence is technically oriented, I believe software improvements are needed to enhance its ability to understand emotions and demonstrate empathy.

**6. Do you have any suggestions for additional questions that should be added to the current ones?**

- How can the balance between artificial intelligence systems and human interaction be maintained? What are the most effective ways to manage interactions between these systems and both clients and professionals?
- What infrastructural changes may be necessary to enable the effective implementation of AI-based systems within healthcare systems?
- What should be considered to make AI systems more accessible and appropriate for children and adolescents from disadvantaged regions?
- What are the long-term effects of AI-based systems on child and adolescent mental health?

**Interview duration:** 45 minutes

**Date:** 21.03.2025

**Participant:PSL-3**

**Name-Surname:** M**A**

**Age:** 32

**Gender:** Female

**How many years have you been working in child and adolescent mental health:** 9

**Title:** Research Assistant / Psychologist /Clinical Psychologist / Assistant Professor / Associate Professor / Professor

**Institution**: Private Practice / State Hospital / University Hospital/Department of Psychology, University/ Ministry of Family and Social Services/School/ Rehabilitation Center

**Have you used ChatGPT before:** Yes

**If yes, for what purposes?** In clinical practice / For writing academic articles / For preparing presentations / For administrative tasks (e.g., tables, annual plans, etc.)

**1.What are your general views on the integration of ChatGPT-4o (an AI-based chatbot) into clinical practice as a child and adolescent mental health professional? What role do you think these tools could play in your profession?**

I believe that the security of information in online environments may pose a barrier to establishing a therapeutic relationship. However, if there is awareness about accessing and filtering accurate information, I think such systems can be functional in terms of generating and accumulating systematic knowledge.

**2.What are the potential advantages and risks of using ChatGPT-4o in the processes of diagnosis, treatment planning, and patient management in child and adolescent mental health?**

If the information is kept up to date, I believe that AI can serve as a supportive tool in the diagnostic process (advantage). However, it may fall short in contributing to recovery, as it would likely miss the nuances of human dynamics, daily life factors, and observational processes in the absence of expert interpretation (disadvantage).

**3.What do you think about the direct use of ChatGPT-4o by child and adolescent clients or their families? Do you believe this tool could be helpful in providing guidance, information, and support, or might it pose risks to the therapeutic process?**

It may provide support for families to practice and develop certain skills. However, just like information found in books, not all content is suitable for every individual or situation. While some people may benefit, others may feel confused or even become discouraged. I believe that AI could offer a contribution similar to that of self-help books, but it will not be effective for everyone.

**4.Do you think ChatGPT-4o could be effective in clinical documentation and medical record management? What are the main practical challenges that could arise from using such systems?**

It can provide support in the systematic recording of information.

**5.What regulations, training, or technical improvements are necessary to ensure more effective use of AI-based systems in child and adolescent mental health in the future? What aspects of ChatGPT-4o should be improved to better support your professional work?**

It can offer support in the areas of integrative evaluation and information analysis based on general data from various administered tests.

**6. Do you have any suggestions for additional questions that should be added to the current ones?**

None.

**Interview duration:** 43 minutes

**Date:** 21.03.2025
