# Supplementary material for: From promise to practice: insights into ChatGPT-4o use in child and adolescent mental health from professionals
Source: Front Psychiatry. 2025 Sep 26;16:1668814. doi: 10.3389/fpsyt.2025.1668814 (PMC12511095; doi:10.3389/fpsyt.2025.1668814)
Supplement: Supplementary file 2 [file DataSheet2.docx]

Supplementary Material-2

**From Promise to Practice: Insights into ChatGPT-4o Use in Child and Adolescent Mental Health from Professionals – Child Psychiatrists' Version – Pilot Study**

This survey has been designed to gather your perspectives on the use of the artificial intelligence-based language model, ChatGPT-4o, within the field of child and adolescent mental health. The questions presented aim to explore your professional experiences, insights, and ethical evaluations regarding this topic.

The survey covers your views on the effectiveness of ChatGPT-4o in clinical practice, its potential areas of application, and its advantages and disadvantages. The data collected will contribute to developing a comprehensive framework regarding the possible role of AI-supported systems in the field of child and adolescent mental health.

This survey is conducted entirely anonymously. No information that could directly identify participants—such as name, surname, email address, or place of employment—is collected.

All responses will be evaluated collectively and used solely for scientific purposes; no individual data will be analyzed separately.

In this study conducted via Google Forms, the "email collection" feature has been disabled, and no link to user accounts is established. Participation is entirely voluntary, and you may withdraw from responding at any time without any consequences.

**Researchers:**
Specialist Dr. Armağan Aral, Assistant Professor Dr. Gizem Gerdan, Associate Professor Dr. Miraç Barış Usta, Specialist Dr. Ayşe Ergüner Aral

**This study has received ethical approval.**
The approval was granted by the İzmir City Hospital Clinical Research Ethics Committee on March 19, 2025 (Approval No: 2025/142).

Completing this survey will take approximately 15 minutes.

Thank you for your participation.

I consent to participate in this study. ☐

Age …………….

Gender

Your title: Resident Doctor/ Specialist Doctor/ Assistant Professor/ Associate Professor/ Professor

Affiliated Institution: Private Hospital / Private Practice/State Hospital/ University Hospital

Have you previously used ChatGPT-4o? Yes/No

For what purposes have you used ChatGPT-4o before? (You may select more than one option.) clinical practice / writing academic papers / preparing presentations / performing administrative tasks (e.g., tables, annual plans, etc.)

How do you think ChatGPT-4o should be appropriately integrated into clinical practice in the field of child and adolescent mental health? (1) ChatGPT-4o has no place in clinical practice, (2) Uncertainty regarding its clinical usefulness, (3) A synergistic effect could emerge by combining mental health professionals' clinical expertise with ChatGPT-4o’s analytical capabilities, and (4) Unquestioning trust in ChatGPT-4o’s diagnostic and treatment suggestions

**Table S2.1. Survey Items Assessing Child and Adolescent Psychiatrists’ Perspectives on ChatGPT-4o Across Thematic Domains**

| Items | Strongly Disagree | Disagree | Neither Agree nor Disagree | Agree | Strongly Agree |
| --- | --- | --- | --- | --- | --- |
| **Profession** |  |  |  |  |  |
| A. Please indicate your level of agreement with each of the following statements regarding the role of ChatGPT-4o in child and adolescent mental health |  |  |  |  |  |
| *A1. It can make diagnostic and treatment decisions without the need for a clinician.* |  |  |  |  |  |
| *A2. It should never replace professional mental health services under any circumstances.* |  |  |  |  |  |
| *A3. It can provide valuable insights by analyzing electronic records, medical literature, and current clinical guidelines.* |  |  |  |  |  |
| *A4. Such tools hold significant potential for advancing professional practices.* |  |  |  |  |  |
| *A5. In the future, it is expected to assume a greater supportive role within clinical practice.* |  |  |  |  |  |
| *A6. It is necessary for us to better adapt to emerging developments in this field.* |  |  |  |  |  |
| **Ethical Issues** |  |  |  |  |  |
| B. Please indicate the appropriate response for each of the following statements regarding your views on the ethical aspects of using ChatGPT-4o in the field of child and adolescent mental health. |  |  |  |  |  |
| *B1. Cases and their families may experience greater concerns regarding confidentiality.* |  |  |  |  |  |
| *B2. The development of ethical guidelines regulating the use of ChatGPT-4o in treatment is essential* |  |  |  |  |  |
| *B3. The use of ChatGPT-4o in clinical practice is unethical.* |  |  |  |  |  |
| **As a Clinician-Facing Tool** |  |  |  |  |  |
| C. Please indicate the appropriate response for each of the following statements regarding your views on the use of ChatGPT-4o as an assistant in clinical practice by mental health professionals. |  |  |  |  |  |
| *C1. Documenting case information into ChatGPT-4o may not be practical.* |  |  |  |  |  |
| *C2. ChatGPT-4o may fail to preserve clinically significant aspects of the original case narrative.* |  |  |  |  |  |
| *C3. It may facilitate the creation of a more systematic case history.* |  |  |  |  |  |
| *C4. It enables case formulations to be carried out more easily.* |  |  |  |  |  |
| *C5. It may help in recalling important points from the case history.* |  |  |  |  |  |
| *C6. Although it may not establish a definitive diagnosis, it can accelerate the diagnostic process.* |  |  |  |  |  |
| *C7. It can generate a complete and accurate list of differential diagnoses even in complex cases.* |  |  |  |  |  |
| *C8. ChatGPT-4o may be beneficial in the use of semi-structured diagnostic interview techniques.* |  |  |  |  |  |
| *C9. It may classify clinically normal conditions as psychiatric diagnoses.* |  |  |  |  |  |
| *C10. It may fail to recognize certain psychiatric diagnoses and lead to missed diagnoses.* |  |  |  |  |  |
| *C11. It has the potential to assist in asking more effective questions to the case and their family.* |  |  |  |  |  |
| *C12. By eliminating nonverbal cues that are crucial in clinical interviews, it may disrupt the therapeutic relationship with the patient and their family.* |  |  |  |  |  |
| *C13. Patients and their families may feel uncomfortable with the delegation of their concerns to an artificial intelligence system, perceiving it as a sign that they are not worthy of human attention.* |  |  |  |  |  |
| *C14. ChatGPT-4o can recognize the predominant mood in the text entered and provide an explanation for it.* |  |  |  |  |  |
| *C15. Clinicians often hinder patients' understanding by overusing technical terms. ChatGPT can be used more effectively in this regard.* |  |  |  |  |  |
| *C16. It provides treatment recommendations based on widely accepted guidelines.* |  |  |  |  |  |
| *C17. It may assist in selecting the appropriate medication.* |  |  |  |  |  |
| *C18. It provides information about possible indications and dosages.* |  |  |  |  |  |
| *C19. The clinician may request detailed information about possible side effects, adverse reactions, laboratory changes, or interactions with other medications.* |  |  |  |  |  |
| *C20. It can be used for skills training and psychoeducation for patients and their families within the framework of Cognitive Behavioral Therapy (CBT).* |  |  |  |  |  |
| *C21. It may demonstrate sufficient creativity in therapy, considering abilities such as storytelling and the use of metaphors.* |  |  |  |  |  |
| *C22. It may assist in the development and implementation of hierarchical exposure tasks in therapeutic settings.* |  |  |  |  |  |
| *C23. It can be used as an aid in assigning behavioral tasks.* |  |  |  |  |  |
| **As a Patient-Facing Tool** |  |  |  |  |  |
| D. Please indicate the appropriate option for each of the following statements regarding your views on the use of ChatGPT-4o by child and adolescents and their families |  |  |  |  |  |
| *D1. The use of ChatGPT-4o may discourage children and adolescents from accessing mental health services when needed.* |  |  |  |  |  |
| *D2. ChatGPT-4o may serve as a tool to offer guidance and support to parents and caregivers in the management of their children’s mental health.* |  |  |  |  |  |
| *D3. Cases and their families should consult such tools before seeing a mental health professional.* |  |  |  |  |  |
| *D4. Such conversational agents can help children, adolescents, and their families better prepare for consultations with psychiatrists or psychologists.* |  |  |  |  |  |
| *D5. ChatGPT-4o saves time by being accessible 24/7 and by answering questions repeatedly without fatigue.* |  |  |  |  |  |
| *D6. While certain diagnostic errors may be inevitable, it is important to understand these errors and how they compare to clinicians’ “gold standard” assessments.* |  |  |  |  |  |
| *D7. Children, adolescents, and their families should have access to relevant, accessible, and timely information regarding mental health status and treatment options in a pressure-free environment, enabling them to make informed health-related decisions freely.* |  |  |  |  |  |
| *D8. ChatGPT-4o has the potential to deliver evidence-based treatment suggestions that are customized according to the individual needs of children, adolescents, and their families.* |  |  |  |  |  |
| *D9. Given that children and adolescents have grown up with such technologies, they may be more predisposed to utilizing tools like ChatGPT-4o to manage psychological difficulties.* |  |  |  |  |  |
| **Acting as a Therapist** |  |  |  |  |  |
| E. Please indicate the appropriate option for each of the following statements regarding the potential roles of ChatGPT-4o as a therapist for children and adolescents. |  |  |  |  |  |
| *E1. As a therapist, ChatGPT-4o is neutral and nonjudgmental; some individuals who fear being judged by a human therapist may find it less intimidating to speak with a machine, thereby expressing sensitive issues more comfortably.* |  |  |  |  |  |
| *E2. Given that human therapists may be influenced by factors such as fatigue, personal life experiences, or their current emotional state, ChatGPT-4o may, in certain situations, offer responses that appear more consistently empathetic.* |  |  |  |  |  |
| *E3. Consistent with approaches like Cognitive Behavioral Therapy (CBT) that focus on self-guided skill acquisition, ChatGPT-4o may offer a range of resources and interactive tools to help individuals develop psychological competencies and understanding.* |  |  |  |  |  |
| *E4. ChatGPT-4o can provide personalized suggestions to support behavioral activation in cases with depressive disorders.* |  |  |  |  |  |
| *E5. ChatGPT-4o can provide personalized recommendations for anger management.* |  |  |  |  |  |
| *E6. ChatGPT-4o can provide personalized recommendations for sleep hygiene.* |  |  |  |  |  |
| *E7. ChatGPT-4o can provide personalized recommendations to help reduce anxiety levels.* |  |  |  |  |  |
| *E8. ChatGPT-4o carries the risk of offering inappropriate advice, which may lead to increased anxiety or even self-harm.* |  |  |  |  |  |
| *E9. ChatGPT-4o may play a supportive role in preventing self-harm. Users may seek assistance from ChatGPT-4o in developing a safety plan aimed at reducing self-injurious behavior.* |  |  |  |  |  |
| **Bias** |  |  |  |  |  |
| F. Please indicate the appropriate option for each of the following statements regarding your views on potential bias in ChatGPT-4o (i.e., displaying certain tendencies or deviating from the principle of neutrality). |  |  |  |  |  |
| *F1. The responses of conversational AI systems are influenced by users’ inputs. ChatGPT-4o generally acknowledges errors and adapts when they are pointed out. However, individuals without the capacity to verify factual accuracy may engage in irrational dialogues, which could pose risks within the field of mental health.* |  |  |  |  |  |
| *F2. Even in the absence of newly introduced information, existing biased content related to mental health—such as the stigmatization of mental disorders, inaccurate media portrayals, discriminatory language, and flawed data—may be used to train language models, thereby perpetuating these biases across digital platforms.* |  |  |  |  |  |
| **General Impressions** |  |  |  |  |  |
| G. Please indicate the appropriate option for each of the following statements regarding your general views on ChatGPT-4o |  |  |  |  |  |
| *G1. These tools offer greater benefits than risks for both cases and their families, as well as for clinicians.* |  |  |  |  |  |
| *G2. It is essential to utilize ChatGPT-4o to enhance the accessibility and quality of mental health services.* |  |  |  |  |  |
| *G3. I believe that gold standard practices and current approaches can be easily integrated into ChatGPT-4o.* |  |  |  |  |  |
| *G4. Entrusting ChatGPT-4o with the assessment of clinical cases involves serious risks.* |  |  |  |  |  |
| *G5. I believe that ChatGPT-4o holds great potential, but it is not yet fully ready for clinical implementation.* |  |  |  |  |  |
| *G6. The use of ChatGPT-4o in clinical practice may contribute to minimizing the rate of medical errors.* |  |  |  |  |  |
| *G7. Relying on ChatGPT-4o in clinical settings may potentially constrain my capacity for independent problem-solving and creative thinking.* |  |  |  |  |  |

**How willing are you to use ChatGPT-4o in your clinical practice?**
*Please rate your willingness on a scale from 1 to 10, where 1 indicates the lowest level of willingness and 10 indicates the highest*

1……………………………………5………………………….10

**Below are some potential areas for improvement in ChatGPT-4o related to child and adolescent mental health.**
*Please select the three features you believe should be prioritized and rank them in order of importance (1 = most important, 2 = second most important, 3 = third most important)*

| Development Area |  | Primary Priority (1) | Secondary Priority (2) | Tertiary Priority (3) |
| --- | --- | --- | --- | --- |
| Ethics | Ethical issues should be addressed through a multidisciplinary approach |  |  |  |
| System Oversight | It is essential that professionals consistently verify and review the content, and that the system is routinely monitored |  |  |  |
| AI Training for Professionals | Mental health professionals should be trained in the use of artificial intelligence software |  |  |  |
| Intelligent Documentation | Software improvements should be made to support documentation processes |  |  |  |
| Clinical Algorithm Training | The software should be trained on diagnostic and treatment algorithms. |  |  |  |
| Psychotherapy Software Training | The software should be trained to support psychotherapy practices |  |  |  |

**Participant Feedback on the Survey**

| Items | Strongly Disagree | Disagree | Neither Agree nor Disagree | Agree | Strongly Agree |
| --- | --- | --- | --- | --- | --- |
| **Please answer the following questions to share your opinions about the survey.** |  |  |  |  |  |
| *The survey questions were clear and easy to understand.* |  |  |  |  |  |
| *The survey was designed to adequately reflect the perspectives of professionals working in child and adolescent mental health.* |  |  |  |  |  |
| *The length and scope of the survey were appropriate.* |  |  |  |  |  |
| *The overall structure of the survey was systematic and logical* |  |  |  |  |  |
| *The survey is likely to provide sufficient data to achieve the research objective.* |  |  |  |  |  |

**Please answer the following questions freely based on your own opinions.**
*When referring to a specific section and question, use the corresponding letter and number code (e.g., use A2 to refer to Section A, Question 2).*

1.Were there any unclear or poorly worded items in the survey? If so, please specify…………………………………………………………………………………………………………………………………………………………………………………………………………………………………

2.Were there any parts of the survey that you found too technical or unnecessary? If yes, please describe. …………………………………………………………………………………………………………………………………………………………………………………………………………………………………

3.Are there any additional topics or items you would recommend including to improve the scope of the survey? …………………………………………………………………………………………………………………………………………………………………………………………………………………………………

4.Are there any ethical, practical, or scientific issues you consider important for clinical practice that were not addressed in the survey? …………………………………………………………………………………………………………………………………………………………………………………………………………………………………

5.Do you have any general comments, suggestions, or criticisms regarding the survey? …………………………………………………………………………………………………………………………………………………………………………………………………………………………………

**From Promise to Practice: Insights into ChatGPT-4o Use in Child and Adolescent Mental Health from Professionals – Psychologist' Version – Pilot Study**

This survey has been designed to gather your perspectives on the use of the artificial intelligence-based language model, ChatGPT-4o, within the field of child and adolescent mental health. The questions presented aim to explore your professional experiences, insights, and ethical evaluations regarding this topic.

The survey covers your views on the effectiveness of ChatGPT-4o in clinical practice, its potential areas of application, and its advantages and disadvantages. The data collected will contribute to developing a comprehensive framework regarding the possible role of AI-supported systems in the field of child and adolescent mental health.

This survey is conducted entirely anonymously. No information that could directly identify participants—such as name, surname, email address, or place of employment—is collected.

All responses will be evaluated collectively and used solely for scientific purposes; no individual data will be analyzed separately.

In this study conducted via Google Forms, the "email collection" feature has been disabled, and no link to user accounts is established. Participation is entirely voluntary, and you may withdraw from responding at any time without any consequences.

**Researchers:**
Specialist Dr. Armağan Aral, Assistant Professor Dr. Gizem Gerdan, Associate Professor Dr. Miraç Barış Usta, Specialist Dr. Ayşe Ergüner Aral

**This study has received ethical approval.**
The approval was granted by the İzmir City Hospital Clinical Research Ethics Committee on March 19, 2025 (Approval No: 2025/142).

Completing this survey will take approximately 15 minutes.

Thank you for your participation.

I consent to participate in this study. ☐

Age …………….

Gender

Your title: Research Assistant/ Psychologist/ Clinical Psychologist / Assistant Professor/ Associate Professor/ Professor

Affiliated Institution: Private Hospital / Private Practice/State Hospital/ University Hospital/Department of Psychology, University/Ministry of Family and Social Services/ School/ Rehabilitation Center

Have you previously used ChatGPT-4o? Yes/No

For what purposes have you used ChatGPT-4o before? (You may select more than one option.) clinical practice / writing academic papers / preparing presentations / performing administrative tasks (e.g., tables, annual plans, etc.)

How do you think ChatGPT-4o should be appropriately integrated into clinical practice in the field of child and adolescent mental health? (1) ChatGPT-4o has no place in clinical practice, (2) Uncertainty regarding its clinical usefulness, (3) A synergistic effect could emerge by combining mental health professionals' clinical expertise with ChatGPT-4o’s analytical capabilities, and (4) Unquestioning trust in ChatGPT-4o’s diagnostic and treatment suggestions

**Table S2.2. Survey Items Assessing Psychologists’ Perspectives on ChatGPT-4o Across Thematic Domains**

| Items | Strongly Disagree | Disagree | Neither Agree nor Disagree | Agree | Strongly Agree |
| --- | --- | --- | --- | --- | --- |
| **Profession** |  |  |  |  |  |
| A. Please indicate your level of agreement with each of the following statements regarding the role of ChatGPT-4o in child and adolescent mental health |  |  |  |  |  |
| *A1. It can make diagnostic and treatment decisions without the need for a clinician.* |  |  |  |  |  |
| *A2. It should never replace professional mental health services under any circumstances.* |  |  |  |  |  |
| *A3. It can provide valuable insights by analyzing electronic records, medical literature, and current clinical guidelines.* |  |  |  |  |  |
| *A4. Such tools hold significant potential for advancing professional practices.* |  |  |  |  |  |
| *A5. In the future, it is expected to assume a greater supportive role within clinical practice.* |  |  |  |  |  |
| *A6. It is necessary for us to better adapt to emerging developments in this field.* |  |  |  |  |  |
| **Ethical Issues** |  |  |  |  |  |
| B. Please indicate the appropriate response for each of the following statements regarding your views on the ethical aspects of using ChatGPT-4o in the field of child and adolescent mental health. |  |  |  |  |  |
| *B1. Cases and their families may experience greater concerns regarding confidentiality.* |  |  |  |  |  |
| *B2. The development of ethical guidelines regulating the use of ChatGPT-4o in treatment is essential* |  |  |  |  |  |
| *B3. The use of ChatGPT-4o in clinical practice is unethical.* |  |  |  |  |  |
| **As a Clinician-Facing Tool** |  |  |  |  |  |
| C. Please indicate the appropriate response for each of the following statements regarding your views on the use of ChatGPT-4o as an assistant in clinical practice by mental health professionals. |  |  |  |  |  |
| *C1. Documenting case information into ChatGPT-4o may not be practical.* |  |  |  |  |  |
| *C2. ChatGPT-4o may fail to preserve clinically significant aspects of the original case narrative.* |  |  |  |  |  |
| *C3. It may facilitate the creation of a more systematic case history.* |  |  |  |  |  |
| *C4. It enables case formulations to be carried out more easily.* |  |  |  |  |  |
| *C5. It may help in recalling important points from the case history.* |  |  |  |  |  |
| *C6. Although it may not establish a definitive diagnosis, it can accelerate the diagnostic process.* |  |  |  |  |  |
| *C7. It can generate a complete and accurate list of differential diagnoses even in complex cases.* |  |  |  |  |  |
| *C8. ChatGPT-4o may be beneficial in the use of semi-structured diagnostic interview techniques.* |  |  |  |  |  |
| *C9. It may classify clinically normal conditions as psychiatric diagnoses.* |  |  |  |  |  |
| *C10. It may fail to recognize certain psychiatric diagnoses and lead to missed diagnoses.* |  |  |  |  |  |
| *C11. It has the potential to assist in asking more effective questions to the case and their family.* |  |  |  |  |  |
| *C12. Patients and their families may feel uncomfortable with the delegation of their concerns to an artificial intelligence system, perceiving it as a sign that they are not worthy of human attention.* |  |  |  |  |  |
| *C13. ChatGPT-4o can recognize the predominant mood in the text entered and provide an explanation for it.* |  |  |  |  |  |
| *C14. Clinicians often hinder patients' understanding by overusing technical terms. ChatGPT can be used more effectively in this regard.* |  |  |  |  |  |
| *C15. It can be used for skills training and psychoeducation for patients and their families within the framework of Cognitive Behavioral Therapy (CBT).* |  |  |  |  |  |
| *C16. It may demonstrate sufficient creativity in therapy, considering abilities such as storytelling and the use of metaphors.* |  |  |  |  |  |
| *C17. It may assist in the development and implementation of hierarchical exposure tasks in therapeutic settings.* |  |  |  |  |  |
| *C18. It can be used as an aid in assigning behavioral tasks.* |  |  |  |  |  |
| **As a Patient-Facing Tool** |  |  |  |  |  |
| D. Please indicate the appropriate option for each of the following statements regarding your views on the use of ChatGPT-4o by child and adolescents and their families |  |  |  |  |  |
| *D1. The use of ChatGPT-4o may discourage children and adolescents from accessing mental health services when needed.* |  |  |  |  |  |
| *D2. ChatGPT-4o may serve as a tool to offer guidance and support to parents and caregivers in the management of their children’s mental health.* |  |  |  |  |  |
| *D3. Cases and their families should consult such tools before seeing a mental health professional.* |  |  |  |  |  |
| *D4. Such conversational agents can help children, adolescents, and their families better prepare for consultations with psychiatrists or psychologists.* |  |  |  |  |  |
| *D5. ChatGPT-4o saves time by being accessible 24/7 and by answering questions repeatedly without fatigue.* |  |  |  |  |  |
| *D6. While certain diagnostic errors may be inevitable, it is important to understand these errors and how they compare to clinicians’ “gold standard” assessments.* |  |  |  |  |  |
| *D7. Children, adolescents, and their families should have access to relevant, accessible, and timely information regarding mental health status and treatment options in a pressure-free environment, enabling them to make informed health-related decisions freely.* |  |  |  |  |  |
| *D8. ChatGPT-4o has the potential to deliver evidence-based treatment suggestions that are customized according to the individual needs of children, adolescents, and their families.* |  |  |  |  |  |
| *D9. Given that children and adolescents have grown up with such technologies, they may be more predisposed to utilizing tools like ChatGPT-4o to manage psychological difficulties.* |  |  |  |  |  |
| **Acting as a Therapist** |  |  |  |  |  |
| E. Please indicate the appropriate option for each of the following statements regarding the potential roles of ChatGPT-4o as a therapist for children and adolescents. |  |  |  |  |  |
| *E1. As a therapist, ChatGPT-4o is neutral and nonjudgmental; some individuals who fear being judged by a human therapist may find it less intimidating to speak with a machine, thereby expressing sensitive issues more comfortably.* |  |  |  |  |  |
| *E2. Given that human therapists may be influenced by factors such as fatigue, personal life experiences, or their current emotional state, ChatGPT-4o may, in certain situations, offer responses that appear more consistently empathetic.* |  |  |  |  |  |
| *E3. Consistent with approaches like Cognitive Behavioral Therapy (CBT) that focus on self-guided skill acquisition, ChatGPT-4o may offer a range of resources and interactive tools to help individuals develop psychological competencies and understanding.* |  |  |  |  |  |
| *E4. ChatGPT-4o can provide personalized suggestions to support behavioral activation in cases with depressive disorders.* |  |  |  |  |  |
| *E5. ChatGPT-4o can provide personalized recommendations for anger management.* |  |  |  |  |  |
| *E6. ChatGPT-4o can provide personalized recommendations for sleep hygiene.* |  |  |  |  |  |
| *E7. ChatGPT-4o can provide personalized recommendations to help reduce anxiety levels.* |  |  |  |  |  |
| *E8. ChatGPT-4o carries the risk of offering inappropriate advice, which may lead to increased anxiety or even self-harm.* |  |  |  |  |  |
| *E9. ChatGPT-4o may play a supportive role in preventing self-harm. Users may seek assistance from ChatGPT-4o in developing a safety plan aimed at reducing self-injurious behavior.* |  |  |  |  |  |
| **Bias** |  |  |  |  |  |
| F. Please indicate the appropriate option for each of the following statements regarding your views on potential bias in ChatGPT-4o (i.e., displaying certain tendencies or deviating from the principle of neutrality). |  |  |  |  |  |
| *F1. The responses of conversational AI systems are influenced by users’ inputs. ChatGPT-4o generally acknowledges errors and adapts when they are pointed out. However, individuals without the capacity to verify factual accuracy may engage in irrational dialogues, which could pose risks within the field of mental health.* |  |  |  |  |  |
| *F2. Even in the absence of newly introduced information, existing biased content related to mental health—such as the stigmatization of mental disorders, inaccurate media portrayals, discriminatory language, and flawed data—may be used to train language models, thereby perpetuating these biases across digital platforms.* |  |  |  |  |  |
| **General Impressions** |  |  |  |  |  |
| G. Please indicate the appropriate option for each of the following statements regarding your general views on ChatGPT-4o |  |  |  |  |  |
| *G1. These tools offer greater benefits than risks for both cases and their families, as well as for clinicians.* |  |  |  |  |  |
| *G2. It is essential to utilize ChatGPT-4o to enhance the accessibility and quality of mental health services.* |  |  |  |  |  |
| *G3. I believe that gold standard practices and current approaches can be easily integrated into ChatGPT-4o.* |  |  |  |  |  |
| *G4. Entrusting ChatGPT-4o with the assessment of clinical cases involves serious risks.* |  |  |  |  |  |
| *G5. I believe that ChatGPT-4o holds great potential, but it is not yet fully ready for clinical implementation.* |  |  |  |  |  |
| *G6. The use of ChatGPT-4o in clinical practice may contribute to minimizing the rate of medical errors.* |  |  |  |  |  |
| *G7. Relying on ChatGPT-4o in clinical settings may potentially constrain my capacity for independent problem-solving and creative thinking.* |  |  |  |  |  |

**How willing are you to use ChatGPT-4o in your clinical practice?**
*Please rate your willingness on a scale from 1 to 10, where 1 indicates the lowest level of willingness and 10 indicates the highest*

1……………………………………5………………………….10

**Below are some potential areas for improvement in ChatGPT-4o related to child and adolescent mental health.**
*Please select the three features you believe should be prioritized and rank them in order of importance (1 = most important, 2 = second most important, 3 = third most important)*

| Development Area |  | Primary Priority (1) | Secondary Priority (2) | Tertiary Priority (3) |
| --- | --- | --- | --- | --- |
| Ethics | Ethical issues should be addressed through a multidisciplinary approach |  |  |  |
| System Oversight | It is essential that professionals consistently verify and review the content, and that the system is routinely monitored |  |  |  |
| AI Training for Professionals | Mental health professionals should be trained in the use of artificial intelligence software |  |  |  |
| Intelligent Documentation | Software improvements should be made to support documentation processes |  |  |  |
| Clinical Algorithm Training | The software should be trained on diagnostic and treatment algorithms. |  |  |  |
| Psychotherapy Software Training | The software should be trained to support psychotherapy practices |  |  |  |

**Participant Feedback on the Survey**

| Items | Strongly Disagree | Disagree | Neither Agree nor Disagree | Agree | Strongly Agree |
| --- | --- | --- | --- | --- | --- |
| **Please answer the following questions to share your opinions about the survey.** |  |  |  |  |  |
| *The survey questions were clear and easy to understand.* |  |  |  |  |  |
| *The survey was designed to adequately reflect the perspectives of professionals working in child and adolescent mental health.* |  |  |  |  |  |
| *The length and scope of the survey were appropriate.* |  |  |  |  |  |
| *The overall structure of the survey was systematic and logical* |  |  |  |  |  |
| *The survey is likely to provide sufficient data to achieve the research objective.* |  |  |  |  |  |

**Please answer the following questions freely based on your own opinions.**
*When referring to a specific section and question, use the corresponding letter and number code (e.g., use A2 to refer to Section A, Question 2).*

1.Were there any unclear or poorly worded items in the survey? If so, please specify…………………………………………………………………………………………………………………………………………………………………………………………………………………………………

2.Were there any parts of the survey that you found too technical or unnecessary? If yes, please describe. …………………………………………………………………………………………………………………………………………………………………………………………………………………………………

3.Are there any additional topics or items you would recommend including to improve the scope of the survey? …………………………………………………………………………………………………………………………………………………………………………………………………………………………………

4.Are there any ethical, practical, or scientific issues you consider important for clinical practice that were not addressed in the survey? …………………………………………………………………………………………………………………………………………………………………………………………………………………………………

5.Do you have any general comments, suggestions, or criticisms regarding the survey? …………………………………………………………………………………………………………………………………………………………………………………………………………………………………
