# Supplementary material for: From promise to practice: insights into ChatGPT-4o use in child and adolescent mental health from professionals
Source: Front Psychiatry. 2025 Sep 26;16:1668814. doi: 10.3389/fpsyt.2025.1668814 (PMC12511095; doi:10.3389/fpsyt.2025.1668814)
Supplement: Supplementary file 3 [file DataSheet3.docx]

Supplementary Material-3

**1. PİLOT STUDY RESULTS FOR CHİLD AND ADOLESCENT PSYCHİATRİSTS AND REVİSİONS**

The preliminary study was conducted with a sample of 5 child and adolescent psychiatrists. The mean age of the participants was 35.6 years, and the group consisted of 3 females and 2 males. In terms of academic rank, four participants were specialists and 1 was an associate professor, all practicing in state hospitals. Regarding prior experience with ChatGPT-4o, 3 participants reported previous use: 2 in clinical practice, 1 during the preparation of an academic manuscript, and 1 for administrative tasks.

Participants were also asked to assess the appropriate positioning of ChatGPT-4o within clinical practice in the field of child and adolescent mental health. Response options included: (1) ChatGPT-4o has no place in clinical practice, (2) Uncertainty regarding its clinical usefulness, (3) A synergistic effect could emerge by combining mental health professionals' clinical expertise with ChatGPT-4o’s analytical capabilities, and (4) Unquestioning trust in ChatGPT-4o’s diagnostic and treatment suggestions. In response, 1participant indicated that ChatGPT-4o has no place in clinical practice, while 4 participants endorsed the view that a synergistic effect between clinical expertise and ChatGPT-4o’s analytical capabilities could be beneficial.

*1.1 Perceptions of ChatGPT-4o’s Profession in Child and Adolescent Mental Health*

Participants were asked to respond to the following items, which assess their perceptions regarding the prfoession of ChatGPT-4o in the field of child and adolescent psychiatry, by selecting the most appropriate option on a 5-point Likert scale (1 = Strongly Disagree, 5 = Strongly Agree) (See Table S2.1 in Suplementary Material 2). The second item was reverse-scored to ensure consistency in interpretation. Following the recoding process, descriptive statistics, including mean scores and standard deviations, were calculated for each item.

**Table S3.1. Perceived Professional Role of ChatGPT-4o: Descriptive Scores**

| Item code | Mean±SD |
| --- | --- |
| A1 | 1.80±0.83 |
| A2 | 2.80±1.64 |
| A3 | 4.00±0.70 |
| A4 | 4.00±1.00 |
| A5 | 4.00±0.70 |
| A6 | 4.40±0.54 |
| **Mean score** | **3.50±0.44** |

Note. Item A2 was reverse-scored.

*1.2 Perceptions of the Ethical Issues of ChatGPT-4o Use in Child and Adolescent Mental Health*

Participants were asked to respond to the following items, which assess their perceptions regarding the ethical issues of ChatGPT-4o in the field of child and adolescent psychiatry, by selecting the most appropriate option on a 5-point Likert scale (1 = Strongly Disagree, 5 = Strongly Agree) (See Table S2.1 in Suplementary Material 2). The items in this section were reverse-coded.

**Table S3.2. Perceived Ethical Issues of ChatGPT-4o: Descriptive Scores**

| Item code | Mean±SD |
| --- | --- |
| B1 | 2.40±1.51 |
| B2 | 1.20±0.44 |
| B3 | 3.40±1.51 |
| **Mean score** | **2.33±0.94** |

Note. All items were reverse-scored

- 1. *Child Psychiatrists' Views on ChatGPT-4o as a Clinician-Facing Tool*

Participants were asked to respond to the following items, which assess their perceptions regarding the clinician-facing tool of ChatGPT-4o in the field of child and adolescent psychiatry, by selecting the most appropriate option on a 5-point Likert scale (1 = Strongly Disagree, 5 = Strongly Agree) (See Table S2.1 in Suplementary Material 2). In the table, items labeled with an "a" suffix (e.g., C1a) were designed to assess participants' beliefs regarding the future resolution of the issues presented in the corresponding primary items. Participants who selected "Agree" or "Strongly Agree" in response to the main item were subsequently asked to respond to these supplementary items. Importantly, the responses to the "a" items were collected for exploratory purposes and were not included in the calculation of subscale or total scores.

**Table S3.3. Perceptions of ChatGPT-4o as a Clinician-Facing Tool: Subscale Scores**

| Item code | Mean±SD |
| --- | --- |
| Documentation (C1-5) |  |
| C1 | 3.40±0.89 |
| C1a | 4.00±1.00 |
| C2 | 2.80±0.83 |
| C2a | 4.00±1.41 |
| C3 | 3.60±1.14 |
| C4 | 3.60±1.14 |
| C5 | 4.20±0.44 |
| **Mean score** | **3.52±0.59** |
| Diagnosis (C6-10) |  |
| C6 | 3.80±0.44 |
| C7 | 3.20±1.30 |
| C8 | 4.00±0.00 |
| C9 | 2.20±0.83 |
| C9a | 4.00±1.41 |
| C10 | 2.00±0.70 |
| C10a | 4.00±1.00 |
| **Mean score** | **3.04±0.62** |
| Therapeutic alliance (C11-15) |  |
| C11 | 3.60±0.54 |
| C12 | 2.20±1.30 |
| C13 | 2.20±0.83 |
| C14 | 3.00±1.22 |
| C15 | 3.20±1.09 |
| **Mean score** | **2.84±0.60** |
| Therapeutic Interventions (C16-19) |  |
| C16 | 4.20±0.44 |
| C17 | 3.80±0.44 |
| C18 | 4.44±0.54 |
| C19 | 4.20±0.44 |
| **Mean score** | **4.15±0.22** |
| CBT-Focused Interventions (C20-23) |  |
| C20 | 3.20±1.30 |
| C21 | 3.60±1.14 |
| C22 | 4.40±0.54 |
| C23 | 4.40±0.54 |
| **Mean score** | **3.90±0.57** |
| **Total mean score** | **3.44±0.40** |

Note: Items C1–2, C9–10, and C12–13 were reverse-scored. Supplementary “a” items were exploratory and not included in score calculations. See Table S2.1, Supplementary File 2, for full item wording.

- 1. *Child Psychiatrists' Views on ChatGPT-4o as a Patient-Facing Tool*

Participants were asked to respond to the following items, which assess their perceptions regarding the patient-facing tool of ChatGPT-4o in the field of child and adolescent psychiatry, by selecting the most appropriate option on a 5-point Likert scale (1 = Strongly Disagree, 5 = Strongly Agree) (See Table S2.1 in Suplementary Material 2).

**Table S3.4. Perceptions of ChatGPT-4o as a Patient-Facing Tool**

| Item code | Mean±SD |
| --- | --- |
| D1 | 3.00±1.58 |
| D2 | 4.20±0.83 |
| D3 | 2.20±0.83 |
| D4 | 3.20±0.83 |
| D5 | 3.80±0.44 |
| D6 | 3.60±0.54 |
| D7 | 3.80±0.44 |
| D8 | 3.60±1.14 |
| D9 | 3.80±0.83 |
| **Mean score** | **3.46±0.64** |

Note. Item D1 was reverse-scored.

*1.5 Views on ChatGPT-4o Acting as a Therapist for Children and Adolescents*

Participants were asked to respond to the following items, which assess their perceptions regarding the acting as a therapist of ChatGPT-4o in the field of child and adolescent psychiatry, by selecting the most appropriate option on a 5-point Likert scale (1 = Strongly Disagree, 5 = Strongly Agree) (See Table S2.1 in Suplementary Material 2).

**Table S3.5. Perceptions of ChatGPT-4o as a Therapist for Children and Adolescents**

| Item code | Mean±SD |
| --- | --- |
| E1 | 3.00±1.14 |
| E2 | 2.60±1.34 |
| E3 | 3.60±1.51 |
| E4 | 3.80±1.09 |
| E5 | 3.80±1.09 |
| E6 | 4.40±0.54 |
| E7 | 3.80±0.83 |
| E8 | 2.80±1.30 |
| E9 | 4.00±0.70 |
| **Mean score** | **3.53±0.87** |

*Note. Item E8 was reverse-scored.*

- 1. *Perceptions of ChatGPT-4o’s Bias and Deviations from Neutrality*

Participants were asked to respond to the following items, which assess their perceptions regarding bias of ChatGPT-4o in the field of child and adolescent psychiatry, by selecting the most appropriate option on a 5-point Likert scale (1 = Strongly Disagree, 5 = Strongly Agree) (See Table S2.1 in Suplementary Material 2). The items in this section were reverse-coded. In the table, items labeled with an "a" suffix (e.g., F1a) were designed to assess participants' beliefs regarding the future resolution of the issues presented in the corresponding primary items. Participants who selected "Agree" or "Strongly Agree" in response to the main item were subsequently asked to respond to these supplementary items. Importantly, the responses to the "a" items were collected for exploratory purposes and were not included in the calculation of subscale or total scores.

**Table S3.6. Perceptions of Bias and Neutrality in ChatGPT-4o**

| Item code | Mean±SD |
| --- | --- |
| F1 | 2.20±1.30 |
| F1a | 3.40±1.51 |
| F2 | 3.00±1.00 |
| F2a | 4.00±0.70 |
| **Mean score** | **2.60±1.14** |

Note. All items were reverse-scored

- 1. *General Impressions of ChatGPT-4o*

Participants were asked to respond to the following items, which assess their general impressions of ChatGPT-4o in the field of child and adolescent psychiatry, by selecting the most appropriate option on a 5-point Likert scale (1 = Strongly Disagree, 5 = Strongly Agree) (See Table S2.1 in Suplementary Material 2).

**Table S3.7. General Impressions of ChatGPT-4o**

| Item code | Mean±SD |
| --- | --- |
| G1 | 3.60±1.51 |
| G2 | 3.60±1.14 |
| G3 | 3.40±1.14 |
| G4 | 2.60±1.51 |
| G5 | 2.00±1.22 |
| G6 | 3.00±1.00 |
| G7 | 3.20±1.30 |
| **Mean score** | **3.05±1.05** |

Note. Items G4,5and 7 were reverse-scored.

Participants indicated their willingness to incorporate ChatGPT-4o into their clinical practice, with a mean score of 6.00 (SD = 3.08). Participants were presented with a list of potential areas for further development of ChatGPT-4o in the field of child and adolescent mental health. They were asked to select the 3 most important features from the list and rank them in order of priority (1 = highest priority, 3 = lowest among the selected). The proposed features included: Ethics (E) — a multidisciplinary approach to ethical issues; System Oversight (SO) — ongoing professional verification and monitoring of system outputs; AI Training for Professionals (ATP) — training mental health professionals in the use of AI tools; Intelligent Documentation (ID) — software improvements for clinical documentation; Clinical Algorithm Training (CAT) — enhancement of diagnostic and treatment algorithms; and Psychotherapy Software Training (PST) — optimization of software to support psychotherapy.

Analysis of participant rankings revealed a notable emphasis on ethical and procedural safeguards. E, S), and ATP were each identified as a primary priority by one participant, indicating a strong shared concern for ensuring responsible and competent use of AI tools in clinical settings. Furthermore, CAT and PST were also selected as primary priorities by other participants, suggesting that the perceived utility of ChatGPT-4o is closely tied to its clinical relevance and integration into therapeutic workflows.

Secondary and tertiary rankings further support this interpretation. CAT and PST received additional secondary and tertiary priority scores, while ID was ranked as a tertiary concern. These patterns suggest that while ethical and oversight mechanisms are viewed as foundational, participants also value functional enhancements that improve AI’s role in diagnosis, documentation, and psychotherapy.

Overall, the diversity of selected priorities reflects a multifaceted perspective among child and adolescent psychiatry professionals: there is clear interest not only in safeguarding practice through ethical and regulatory mechanisms, but also in enhancing practical applications of AI to improve clinical efficiency and therapeutic effectiveness.

**Table S3.8. Participant Prioritization of ChatGPT-4o Development Areas in Child and Adolescent Mental Health**

| Development Area | Abbreviation | Primary Priority (1) | Secondary Priority (2) | Tertiary Priority (3) |
| --- | --- | --- | --- | --- |
| Ethics | E | 1 |  |  |
| System Oversight | SO | 1 |  |  |
| AI Training for Professionals | ATP | 1 |  |  |
| Intelligent Documentation | ID |  |  | 3 |
| Clinical Algorithm Training | CAT | 1 | 3 | 2 |
| Psychotherapy Software Training | PST | 1 | 2 |  |

Note. Participants were asked to select and rank the three most important areas for future development of ChatGPT-4o in the context of child and adolescent psychiatry. Rankings reflect the perceived relative priority of each feature (1 = highest).

*1.8 Participant Feedback on the Survey*

At the conclusion of the pilot study, participants were invited to provide feedback regarding the survey's clarity, content relevance, length and comprehensiveness, structural coherence, and data adequacy. Their evaluations offer valuable insights into the survey’s design quality and its alignment with the research objectives. The feedback serves not only as an assessment of the instrument’s initial validity but also as a guide for potential refinements before broader implementation.

**Table S3.9. Participant Feedback after Pilot Study**

| Variables | Mean±SD |
| --- | --- |
| Clarity | 4.20±0.83 |
| Content Suitability | 4.40±0.54 |
| Length and Comprehensiveness | 3.60±1.51 |
| Structural Coherence | 4.40±0.54 |
| Data Adequacy | 4.40±0.54 |
| **Mean score** | **4.20±0.67** |

Note. **Clarity:** The survey questions were clearly and understandably formulated; **Content Suitability:** The survey was designed to adequately capture the perspectives of child and adolescent mental health professionals; **Length and Comprehensiveness:** The survey’s length and scope were deemed appropriate; **Structural Coherence:** The survey’s overall structure demonstrated systematic organization and logical coherence;**Data Adequacy**: The survey was designed to yield sufficient data to meet the research aims

*1.9 Item Selection*

In the initial phase of survey development, a pilot study was conducted to evaluate item clarity, internal consistency, and variability in participant responses. Items with mean scores below 3.00 and standard deviations exceeding 1.20 were identified as candidates for removal, in line with recommendations from previous psychometric research emphasizing the importance of item discrimination and reliability (1, 2). Specifically, the following items were excluded due to low endorsement or high variability, suggesting poor interpretability or conceptual ambiguity: A2, B3, C2, C9, C10, C12, C13, D3, E2, E8, F1, G4, and G5. The removal of reverse-coded items with low scores also aimed to minimize response burden and reduce cognitive load, which are known to impact the validity of self-report measures (3).

Moreover, items that displayed semantic overlap with others or elicited redundant response patterns were also considered for exclusion. For instance, items C9 and C10 addressed similar diagnostic risks and demonstrated both low means and high standard deviations, indicating weak item-level contribution to the overall construct. This step aligns with best practices in questionnaire refinement, which recommend parsimony and clarity in scale construction to enhance factorial integrity and respondent engagement (4). Following item reduction, the revised version of the scale retained a more balanced distribution of item difficulty and improved the instrument’s conceptual coherence.

In addition to item-level statistical criteria, qualitative feedback and semantic clarity were also considered during the item refinement process. Item D6 (*“While certain diagnostic errors may be inevitable, it is important to understand these errors and how they compare to clinicians’ ‘gold standard’ assessments”*) was excluded due to its complex wording and abstract structure, which could hinder comprehension. The item presents a conditional clause followed by a comparative evaluation, requiring high cognitive load from respondents—especially in a self-administered format. According to best practices in scale development, survey items should be formulated in a concise and unambiguous manner to reduce respondent fatigue and enhance response validity (1); complex or double-barreled items may compromise content validity and introduce measurement error (5). Additionally, pilot participants did not provide consistent responses to this item, suggesting interpretive ambiguity. Therefore, item D6 was removed to maintain the clarity and psychometric integrity of the final scale.

During the item refinement phase, F2 (*“Even in the absence of newly introduced information, existing biased content related to mental health—such as the stigmatization of mental disorders, inaccurate media portrayals, discriminatory language, and flawed data—may be used to train language models, thereby perpetuating these biases across digital platforms”*) was identified as semantically dense and syntactically complex. The item contained multiple embedded clauses and examples within a single sentence, increasing cognitive load and potentially impairing comprehension. In line with survey design guidelines, overly lengthy or multi-concept items are discouraged, as they can compromise both content validity and the reliability of participant responses (5). Consequently, the item was revised into a more concise and accessible format, preserving its conceptual essence while enhancing clarity and interpretability for respondents.

**References**

1. DeVellis RF, Thorpe CT. Scale development: Theory and applications: Sage publications; 2021.

2. Boateng GO, Neilands TB, Frongillo EA, Melgar-Quiñonez HR, Young SL. Best practices for developing and validating scales for health, social, and behavioral research: a primer. Frontiers in public health. 2018;6:149.

3. Weijters B, Baumgartner H. Misresponse to reversed and negated items in surveys: A review. Journal of Marketing Research. 2012;49(5):737-747.

4. Worthington RL, Whittaker TA. Scale development research: A content analysis and recommendations for best practices. The counseling psychologist. 2006;34(6):806-838.

5. Fowler Jr FJ. Survey research methods: Sage publications; 2013.

**2. PİLOT STUDY RESULTS FOR PSYCHOLOGİSTS AND REVİSİONS**

The preliminary study was conducted with a sample of 5 psychologist. The mean age of the participants was 31.2 years, and the group consisted of 5 females. In terms of academic rank, 4 participants were clinical psychologists and 1 was an assistant professor, all practicing in private psychology clinic. Regarding prior experience with ChatGPT-4o, all participants reported previous use: 3 in clinical practice, 1 during the preparation of an academic manuscript, and 1 for administrative tasks.

Participants were also asked to assess the appropriate positioning of ChatGPT-4o within clinical practice in the field of child and adolescent mental health. Response options included: (1) ChatGPT-4o has no place in clinical practice, (2) Uncertainty regarding its clinical usefulness, (3) A synergistic effect could emerge by combining mental health professionals' clinical expertise with ChatGPT-4o’s analytical capabilities, and (4) Unquestioning trust in ChatGPT-4o’s diagnostic and treatment suggestions. In response, 1 participant stated that ChatGPT-4o has no place in clinical practice, 2 expressed uncertainty, and 2 participants endorsed the potential for a synergistic collaboration between clinical expertise and ChatGPT-4o’s analytical abilities.

*2.1 Perceptions of ChatGPT-4o’s Profession in Child and Adolescent Mental Health*

Participants were asked to respond to the following items, which assess their perceptions regarding the prfoession of ChatGPT-4o in the field of child and adolescent mental health, by selecting the most appropriate option on a 5-point Likert scale (1 = Strongly Disagree, 5 = Strongly Agree) (See Table S2.2 in Suplementary Material 2). The second item was reverse-scored to ensure consistency in interpretation. Following the recoding process, descriptive statistics, including mean scores and standard deviations, were calculated for each item.

**Table S3.10. Perceived Professional Role of ChatGPT-4o: Descriptive Scores**

| Item code | Mean±SD |
| --- | --- |
| A1 | 1.20±0.44 |
| A2 | 1.60±1.34 |
| A3 | 3.60±1.14 |
| A4 | 3.00±1.00 |
| A5 | 3.60±0.54 |
| A6 | 3.80±0.83 |
| **Mean score** | **2.80±0.70** |

Note. Item A2 was reverse-scored.

*2.2 Perceptions of the Ethical Issues of ChatGPT-4o Use in Child and Adolescent Mental Health*

Participants were asked to respond to the following items, which assess their perceptions regarding the ethical issues of ChatGPT-4o in the field of child and adolescent mental health, by selecting the most appropriate option on a 5-point Likert scale (1 = Strongly Disagree, 5 = Strongly Agree) (See Table S2.2 in Suplementary Material 2). The items in this section were reverse-coded.

**Table S3.11. Perceived Ethical Issues of ChatGPT-4o: Descriptive Scores**

| Item code | Mean±SD |
| --- | --- |
| B1 | 1.80±0.83 |
| B2 | 1.60±0.89 |
| B3 | 2.80±1.09 |
| **Mean score** | **2.06±0.76** |

*Note. All items were reverse-coded*

*2.3 Psychologists' Views on ChatGPT-4o as a Clinician-Facing Tool*

Participants were asked to respond to the following items, which assess their perceptions regarding the clinician-facing tool of ChatGPT-4o in the field of child and adolescent mental health, by selecting the most appropriate option on a 5-point Likert scale (1 = Strongly Disagree, 5 = Strongly Agree) (See Table S2.2 in Suplementary Material 2). In the table, items labeled with an "a" suffix (e.g., C1a) were designed to assess participants' beliefs regarding the future resolution of the issues presented in the corresponding primary items. Participants who selected "Agree" or "Strongly Agree" in response to the main item were subsequently asked to respond to these supplementary items. Importantly, the responses to the "a" items were collected for exploratory purposes and were not included in the calculation of subscale or total scores.

**Table S3.12. Perceptions of ChatGPT-4o as a Clinician-Facing Tool: Subscale Scores**

| Item code | Mean±SD |
| --- | --- |
| Documentation (C1-5) |  |
| C1 | 3.20±0.83 |
| C1a | 3.25±0.95 |
| C2 | 2.40±0.54 |
| C2a | 3.25±0.95 |
| C3 | 3.20±1.09 |
| C4 | 3.40±0.89 |
| C5 | 3.40±0.89 |
| **Mean score** | **3.12±0.67** |
| Diagnosis (C6-10) |  |
| C6 | 3.00±0.70 |
| C7 | 2.80±0.83 |
| C8 | 3.00±0.70 |
| C9 | 3.00±1.00 |
| C9a | 3.50±1.29 |
| C10 | 2.00±0.70 |
| C10a | 3.25±0.95 |
| **Mean score** | **2.76±0.38** |
| Therapeutic alliance (C11-14) |  |
| C11 | 3.40±0.89 |
| C12 | 2.00±0.70 |
| C13 | 3.00±0.70 |
| C14 | 3.20±1.30 |
| **Mean score** | **2.90±0.62** |
| Therapeutic Interventions (C15-18) |  |
| C15 | 2.80±1.30 |
| C16 | 3.20±0.83 |
| C17 | 3.60±0.54 |
| C18 | 3.60±0.54 |
| **Mean score** | **3.30±0.67** |
| **Total mean score** | **3.01±0.49** |

Note: Items C1–2, C9–10, and C12 were reverse-scored. Supplementary “a” items were exploratory and not included in score calculations. See Table S2.2, Supplementary File 2, for full item wording.

*2.4 Psychologists' Views on ChatGPT-4o as a Patient-Facing Tool*

Participants were asked to respond to the following items, which assess their perceptions regarding the patient-facing tool of ChatGPT-4o in the field of child and adolescent mental health, by selecting the most appropriate option on a 5-point Likert scale (1 = Strongly Disagree, 5 = Strongly Agree) (See Table S2.2 in Suplementary Material 2).

**Table S3.13. Perceptions of ChatGPT-4o as a Patient-Facing Tool**

| Item code | Mean±SD |
| --- | --- |
| D1 | 2.20±0.44 |
| D2 | 3.40±0.89 |
| D3 | 2.00±1.00 |
| D4 | 2.40±1.34 |
| D5 | 2.40±1.34 |
| D6 | 4.00±0.70 |
| D7 | 3.40±1.51 |
| D8 | 2.60±1.51 |
| D9 | 3.40±1.34 |
| **Mean score** | **2.86±0.76** |

Note. Item D1 was reverse-scored

*2.5 Views on ChatGPT-4o Acting as a Therapist for Children and Adolescents*

Participants were asked to respond to the following items, which assess their perceptions regarding the acting as a therapist of ChatGPT-4o in the field of child and adolescent mental health, by selecting the most appropriate option on a 5-point Likert scale (1 = Strongly Disagree, 5 = Strongly Agree) (See Table S2.2 in Suplementary Material 2).

**Table S3.14. Perceptions of ChatGPT-4o as a Therapist for Children and Adolescents**

| Item code | Mean±SD |
| --- | --- |
| E1 | 3.00±1.41 |
| E2 | 2.60±1.51 |
| E3 | 3.40±1.51 |
| E4 | 3.20±1.30 |
| E5 | 3.60±0.89 |
| E6 | 3.80±0.44 |
| E7 | 3.80±0.44 |
| E8 | 3.00±1.22 |
| E9 | 3.20±0.83 |
| **Mean score** | **328.±0.91** |

Note. Item E8 was reverse-scored.

- 1. *Perceptions of ChatGPT-4o’s Bias and Deviations from Neutrality*

Participants were asked to respond to the following items, which assess their perceptions regarding bias of ChatGPT-4o in the field of child and adolescent mental health, by selecting the most appropriate option on a 5-point Likert scale (1 = Strongly Disagree, 5 = Strongly Agree) (See Table S2.2 in Suplementary Material 2). The items in this section were reverse-coded. In the table, items labeled with an "a" suffix (e.g., F1a) were designed to assess participants' beliefs regarding the future resolution of the issues presented in the corresponding primary items. Participants who selected "Agree" or "Strongly Agree" in response to the main item were subsequently asked to respond to these supplementary items. Importantly, the responses to the "a" items were collected for exploratory purposes and were not included in the calculation of subscale or total scores.

**Table S3.15. Perceptions of Bias and Neutrality in ChatGPT-4o**

| Item code | Mean±SD |
| --- | --- |
| F1 | 1.60±0.54 |
| F1a | 3.20±0.83 |
| F2 | 2.00±1.22 |
| F2a | 3.00±0.70 |
| **Mean score** | **1.80±0.83** |

Note. All items were reverse-scored

- 1. *General Impressions of ChatGPT-4o*

Participants were asked to respond to the following items, which assess their general impressions of ChatGPT-4o in the field of child and adolescent mental health, by selecting the most appropriate option on a 5-point Likert scale (1 = Strongly Disagree, 5 = Strongly Agree) (See Table S2.2 in Suplementary Material 2).

**Table S3.16. General Impressions of ChatGPT-4o**

| Item code | Mean±SD |
| --- | --- |
| G1 | 2.60±1.14 |
| G2 | 3.00±1.22 |
| G3 | 2.80±1.30 |
| G4 | 2.20±1.30 |
| G5 | 2.20±1.09 |
| G6 | 2.80±0.83 |
| G7 | 3.60±1.34 |
| **Mean score** | **2.74±0.75** |

Note. Items G4,5and 7 were reverse-scored.

Participants indicated their willingness to incorporate ChatGPT-4o into their clinical practice, with a mean score of 5.00 (SD = 2.00). Participants were presented with a list of potential areas for further development of ChatGPT-4o in the field of child and adolescent mental health. They were asked to select the 3 most important features from the list and rank them in order of priority (1 = highest priority, 3 = lowest among the selected). The proposed features included: Ethics (E) — a multidisciplinary approach to ethical issues; System Oversight (SO) — ongoing professional verification and monitoring of system outputs; AI Training for Professionals (ATP) — training mental health professionals in the use of AI tools; Intelligent Documentation (ID) — software improvements for clinical documentation; Clinical Algorithm Training (CAT) — enhancement of diagnostic and treatment algorithms; and Psychotherapy Software Training (PST) — optimization of software to support psychotherapy.

Analysis of participant rankings revealed a notable emphasis on ethical and procedural safeguards. E, SO, and ATP were each identified as a primary priority by one participant, indicating a strong shared concern for ensuring responsible and competent use of AI tools in clinical settings. Furthermore, CAT and PST were also selected as primary priorities by other participants, suggesting that the perceived utility of ChatGPT-4o is closely tied to its clinical relevance and integration into therapeutic workflows.

Secondary and tertiary rankings further support this interpretation. CAT and PST received additional secondary and tertiary priority scores, while ID was ranked as a tertiary concern. These patterns suggest that while ethical and oversight mechanisms are viewed as foundational, participants also value functional enhancements that improve AI’s role in diagnosis, documentation, and psychotherapy.

Overall, the diversity of selected priorities reflects a multifaceted perspective among child and adolescent psychiatry professionals: there is clear interest not only in safeguarding practice through ethical and regulatory mechanisms, but also in enhancing practical applications of AI to improve clinical efficiency and therapeutic effectiveness.

**Table S3.17. Participant Prioritization of ChatGPT-4o Development Areas in Child and Adolescent Mental Health**

| Development Area | Abbreviation | Primary Priority (1) | Secondary Priority (2) | Tertiary Priority (3) |
| --- | --- | --- | --- | --- |
| Ethics | E | 4 | 1 |  |
| System Oversight | SO | 1 |  | 4 |
| AI Training for Professionals | ATP |  | 3 |  |
| Intelligent Documentation | ID |  |  |  |
| Clinical Algorithm Training | CAT |  | 1 | 1 |
| Psychotherapy Software Training | PST |  |  |  |

Note. Participants were asked to select and rank the three most important areas for future development of ChatGPT-4o in the context of child and adolescent psychiatry. Rankings reflect the perceived relative priority of each feature (1 = highest).

*2.8 Item selection*

In the pilot phase of the study, descriptive item analysis was conducted based on mean and standard deviation values to evaluate the clarity, discriminatory power, and necessity of each item. Items with extremely low standard deviation (e.g., A1: M=1.20, SD=0.44; B2: M=1.60, SD=0.89; D1: M=2.20, SD=0.44; F1: M=1.60, SD=0.54) indicated limited variability and thus low discriminatory capacity, suggesting a strong consensus among participants regardless of individual differences. Consistent with established item development guidelines, items exhibiting minimal variation and highly skewed response distributions are typically removed as they do not contribute meaningfully to the scale’s sensitivity or construct validity (1).

Additionally, reverse-coded items (e.g., D1, E8, G4,G5,G7) were reviewed for internal consistency. While reverse items are often used to control for response bias, their inclusion in small-scale pilot studies can lead to confusion and reduce reliability if participants interpret them inconsistently (6). Given that several of these reverse-coded items received highly variable responses (e.g., G4-5: SD=1.30; E8: SD=1.22), they were considered for revision or removal. Taken together, this item reduction process ensured that only clear, relevant, and psychometrically robust items were retained for the final version of the survey instrument.

In addition to item-level statistical criteria, qualitative feedback and semantic clarity were also considered during the item refinement process. Item D6 (*“While certain diagnostic errors may be inevitable, it is important to understand these errors and how they compare to clinicians’ ‘gold standard’ assessments”*) was excluded due to its complex wording and abstract structure, which could hinder comprehension. The item presents a conditional clause followed by a comparative evaluation, requiring high cognitive load from respondents—especially in a self-administered format. According to best practices in scale development, survey items should be formulated in a concise and unambiguous manner to reduce respondent fatigue and enhance response validity (1); complex or double-barreled items may compromise content validity and introduce measurement error (5). Additionally, pilot participants did not provide consistent responses to this item, suggesting interpretive ambiguity. Therefore, item D6 was removed to maintain the clarity and psychometric integrity of the final scale.

During the item refinement phase, F2 (*“Even in the absence of newly introduced information, existing biased content related to mental health—such as the stigmatization of mental disorders, inaccurate media portrayals, discriminatory language, and flawed data—may be used to train language models, thereby perpetuating these biases across digital platforms”*) was identified as semantically dense and syntactically complex. The item contained multiple embedded clauses and examples within a single sentence, increasing cognitive load and potentially impairing comprehension. In line with survey design guidelines, overly lengthy or multi-concept items are discouraged, as they can compromise both content validity and the reliability of participant responses (3). Consequently, the item was revised into a more concise and accessible format, preserving its conceptual essence while enhancing clarity and interpretability for respondents.

**References**

1. DeVellis RF, Thorpe CT. Scale development: Theory and applications: Sage publications; 2021.

2. Boateng GO, Neilands TB, Frongillo EA, Melgar-Quiñonez HR, Young SL. Best practices for developing and validating scales for health, social, and behavioral research: a primer. Frontiers in public health. 2018;6:149.

3. Weijters B, Baumgartner H. Misresponse to reversed and negated items in surveys: A review. Journal of Marketing Research. 2012;49(5):737-747.

4. Worthington RL, Whittaker TA. Scale development research: A content analysis and recommendations for best practices. The counseling psychologist. 2006;34(6):806-838.

5. Fowler Jr FJ. Survey research methods: Sage publications; 2013.

6. Swain SD, Weathers D, Niedrich RW. Assessing three sources of misresponse to reversed Likert items. Journal of marketing research. 2008;45(1):116-131.
