# Supplementary material for: From promise to practice: insights into ChatGPT-4o use in child and adolescent mental health from professionals
Source: Front Psychiatry. 2025 Sep 26;16:1668814. doi: 10.3389/fpsyt.2025.1668814 (PMC12511095; doi:10.3389/fpsyt.2025.1668814)
Supplement: Supplementary file 4 [file DataSheet4.docx]

Supplementary Material-4

**Table S4.1.** **Survey Items Regarding the Views on the Role of ChatGPT-4o in Clinical Practice for Child and Adolescent Pschiatrists**

| **Subscale** | **Subsection** | **Subsection Theme** | **Items** | Rate the below items as: 1=Strongly Disagree, 2=Disagree, 3=Uncertainty, 4=Agree, 5=Strongly Agree | |
| --- | --- | --- | --- | --- | --- |
| A. Please indicate your level of agreement with each of the following statements regarding the role of ChatGPT-4o in child and adolescent mental health | | | | | |
| **Profession** |  |  | *A1. It can make diagnostic and treatment decisions without the need for a clinician.* |  |  |
|  |  |  | *A2. It can provide valuable insights by analyzing electronic records, medical literature, and current clinical guidelines.* |  |  |
|  |  |  | *A3. Such tools hold significant potential for advancing professional practices.* |  |  |
|  |  |  | *A4. In the future, it is expected to assume a greater supportive role within clinical practice.* |  |  |
|  |  |  | *A5. It is necessary for us to better adapt to emerging developments in this field.* |  |  |
| B. Please indicate the appropriate response for each of the following statements regarding your views on the ethical aspects of using ChatGPT-4o in the field of child and adolescent mental health. | | | | | |
| **Ethical Issues** |  |  | *B1. Cases and their families may experience greater concerns regarding confidentiality.* |  |  |
|  |  |  | *B2. The development of ethical guidelines regulating the use of ChatGPT-4o in treatment is essential* |  |  |
| C. Please indicate the appropriate response for each of the following statements regarding your views on the use of ChatGPT-4o as an assistant in clinical practice by mental health professionals. | | | | | |
| **As a Clinician-Facing Tool** | **Documentation and Case Formulation** |  | *C1. Documenting case information into ChatGPT-4o may not be practical.* |  |  |
|  |  |  | *C2. It may facilitate the creation of a more systematic case history.* |  |  |
|  |  |  | *C3. It enables case formulations to be carried out more easily.* |  |  |
|  |  |  | *C4. It may help in recalling important points from the case history.* |  |  |
|  |  |  | *C5. It has the potential to assist in asking more effective questions to the case and their family.* |  |  |
|  |  |  | *C6. ChatGPT-4o can recognize the predominant mood in the text entered and provide an explanation for it.* |  |  |
|  | **Clinical Diagnosis** |  | *C7. Although it may not establish a definitive diagnosis, it can accelerate the diagnostic process.* |  |  |
|  |  |  | *C8. It can generate a complete and accurate list of differential diagnoses even in complex cases.* |  |  |
|  |  |  | *C9. ChatGPT-4o may be beneficial in the use of semi-structured diagnostic interview techniques.* |  |  |
|  | **Treatment** | **Psychoeducation** | *C10. Clinicians often hinder patients' understanding by overusing technical terms. ChatGPT can be used more effectively in this regard.* |  |  |
|  |  | **Management** | *C11. It provides treatment recommendations based on widely accepted guidelines.* |  |  |
|  |  | **Medication Guidance** | *C12. It may assist in selecting the appropriate medication.* |  |  |
|  |  |  | *C13. It provides information about possible indications and dosages.* |  |  |
|  |  |  | *C14. The clinician may request detailed information about possible side effects, adverse reactions, laboratory changes, or interactions with other medications.* |  |  |
|  |  | **Structured Psychotherapeutic Support** | *C15. It can be used for skills training and psychoeducation for patients and their families within the framework of Cognitive Behavioral Therapy (CBT).* |  |  |
|  |  |  | *C16. It may demonstrate sufficient creativity in therapy, considering abilities such as storytelling and the use of metaphors.* |  |  |
|  |  |  | *C17. It may assist in the development and implementation of hierarchical exposure tasks in therapeutic settings.* |  |  |
|  |  |  | *C18. It can be used as an aid in assigning behavioral tasks.* |  |  |
| D. Please indicate the appropriate option for each of the following statements regarding your views on the use of ChatGPT-4o by child and adolescents and their families | | | | | |
| **As a Patient-Facing Tool** | **Risks of Independent Use** |  | *D1. The use of ChatGPT-4o may discourage children and adolescents from accessing mental health services when needed.* |  |  |
|  | **Psychoeducational Support for Families** |  | *D2. ChatGPT-4o may serve as a tool to offer guidance and support to parents and caregivers in the management of their children’s mental health.* |  |  |
|  |  |  | *D3. Such conversational agents can help children, adolescents, and their families better prepare for consultations with psychiatrists or psychologists.* |  |  |
|  |  |  | *D4. Children, adolescents, and their families should have access to relevant, accessible, and timely information regarding mental health status and treatment options in a pressure-free environment, enabling them to make informed health-related decisions freely* |  |  |
|  | **Digital Access and Personalization in Youth Mental Health** |  | *D5. ChatGPT-4o saves time by being accessible 24/7 and by answering questions repeatedly without fatigue* |  |  |
|  |  |  | *D6. ChatGPT-4o has the potential to deliver evidence-based treatment suggestions that are customized according to the individual needs of children, adolescents, and their families.* |  |  |
|  |  |  | *D7. Given that children and adolescents have grown up with such technologies, they may be more predisposed to utilizing tools like ChatGPT-4o to manage psychological difficulties.* |  |  |
| E. Please indicate the appropriate option for each of the following statements regarding the potential roles of ChatGPT-4o as a therapist for children and adolescents | | | | | |
| **Acting as a Therapist** | **Therapeutic Alliance** |  | *E1. As a therapist, ChatGPT-4o is neutral and nonjudgmental; some individuals who fear being judged by a human therapist may find it less intimidating to speak with a machine, thereby expressing sensitive issues more comfortably.* |  |  |
|  | **Self-Help and Behavior Change Interventions** |  | *E2. Consistent with approaches like Cognitive Behavioral Therapy (CBT) that focus on self-guided skill acquisition, ChatGPT-4o may offer a range of resources and interactive tools to help individuals develop psychological competencies and understanding.* |  |  |
|  |  |  | *E3. ChatGPT-4o can provide personalized suggestions to support behavioral activation in cases with depressive disorders.* |  |  |
|  |  |  | *E4. ChatGPT-4o can provide personalized recommendations for anger management.* |  |  |
|  |  |  | *E5. ChatGPT-4o can provide personalized recommendations for sleep hygiene.* |  |  |
|  |  |  | *E6. ChatGPT-4o can provide personalized recommendations to help reduce anxiety levels.* |  |  |
|  | **Crisis Prevention and Safety Planning** |  | *E7. ChatGPT-4o may play a supportive role in preventing self-harm. Users may seek assistance from ChatGPT-4o in developing a safety plan aimed at reducing self-injurious behavior.* |  |  |
| F. Please indicate the appropriate option for each of the following statements regarding your views on potential bias in ChatGPT-4o (i.e., displaying certain tendencies or deviating from the principle of neutrality). | | | | | |
| **Bias** |  |  | *F1. Even in the absence of newly introduced information, existing biased content related to mental health—such as the stigmatization of mental disorders, inaccurate media portrayals, discriminatory language, and flawed data—may be used to train language models, thereby perpetuating these biases across digital platforms.* |  |  |
| G. Please indicate the appropriate option for each of the following statements regarding your general views on ChatGPT-4o | | | | | |
| **General Impressions** |  |  | *G1. It is essential to utilize ChatGPT-4o to enhance the accessibility and quality of mental health services.* |  |  |
|  |  |  | *G2. I believe that gold standard practices and current approaches can be easily integrated into ChatGPT-4o.* |  |  |
|  |  |  | *G3. The use of ChatGPT-4o in clinical practice may contribute to minimizing the rate of medical errors.* |  |  |
|  |  |  | *G4. Relying on ChatGPT-4o in clinical settings may potentially constrain my capacity for independent problem-solving and creative thinking.* |  |  |
| **Willingness to Use ChatGPT-4o** |  |  | *H. How willing are you to use ChatGPT-4o in your clinical practice?* | *Please rate your willingness on a scale from 1 to 10, where 1 indicates the lowest level of willingness and 10 indicates the highest* | |
| I. Below are some potential areas for improvement in ChatGPT-4o related to child and adolescent mental health.  *Please rate the importance of each feature for you on a scale from 1 to 10 (1 = not important at all, 10 = extremely important).* | | | | | |
| **AI Development Priorities** |  |  | *Ethical* | Ethical issues should be addressed through a multidisciplinary approach | |
|  |  |  | *System Oversight* | It is essential that professionals consistently verify and review the content, and that the system is routinely monitored | |
|  |  |  | *AI Training for Professionals* | Mental health professionals should be trained in the use of artificial intelligence software | |
|  |  |  | *Software – Diagnostic & Treatment Support* | Mental health professionals should be trained in the use of artificial intelligence software | |
|  |  |  | *Software – Psychotherapy Support* | The software should be trained to support psychotherapy practices | |

**Table S4.2.** **Survey Items Regarding the Views on the Role of ChatGPT-4o in Clinical Practice for Psychologists**

| **Subscale** | **Subsection** | **Subsection Theme** | **Items** | Rate the below items as: 1=Strongly Disagree, 2=Disagree, 3=Uncertainty, 4=Agree, 5=Strongly Agree | |
| --- | --- | --- | --- | --- | --- |
| A. Please indicate your level of agreement with each of the following statements regarding the role of ChatGPT-4o in child and adolescent mental health | | | | | |
| **Profession** |  |  | *A1. It should never replace professional mental health services under any circumstances.* |  |  |
|  |  |  | *A2. It can provide valuable insights by analyzing electronic records, medical literature, and current clinical guidelines.* |  |  |
|  |  |  | *A3. Such tools hold significant potential for advancing professional practices.* |  |  |
|  |  |  | *A4. In the future, it is expected to assume a greater supportive role within clinical practice.* |  |  |
|  |  |  | *A5. It is necessary for us to better adapt to emerging developments in this field.* |  |  |
| B. Please indicate the appropriate response for each of the following statements regarding your views on the ethical aspects of using ChatGPT-4o in the field of child and adolescent mental health. | | | | | |
| **Ethical Issues** |  |  | *B1. Cases and their families may experience greater concerns regarding confidentiality.* |  |  |
|  |  |  | *B2. The use of ChatGPT-4o in clinical practice is unethical.* |  |  |
| C. Please indicate the appropriate response for each of the following statements regarding your views on the use of ChatGPT-4o as an assistant in clinical practice by mental health professionals. | | | | | |
| **As a Clinician-Facing Tool** | **Documentation and Case Formulation** |  | *C1. Documenting case information into ChatGPT-4o may not be practical.* |  |  |
|  |  |  | *C2. ChatGPT-4o may fail to preserve clinically significant aspects of the original case narrative.* |  |  |
|  |  |  | *C3. It may facilitate the creation of a more systematic case history.* |  |  |
|  |  |  | *C4. It enables case formulations to be carried out more easily.* |  |  |
|  |  |  | *C5. It may help in recalling important points from the case history.* |  |  |
|  |  |  | *C6. It has the potential to assist in asking more effective questions to the case and their family.* |  |  |
|  |  |  | *C7. ChatGPT-4o can recognize the predominant mood in the text entered and provide an explanation for it.* |  |  |
|  | **Clinical Diagnosis** |  | *C8. Although it may not establish a definitive diagnosis, it can accelerate the diagnostic process.* |  |  |
|  |  |  | *C9. It can generate a complete and accurate list of differential diagnoses even in complex cases.* |  |  |
|  |  |  | *C10. ChatGPT-4o may be beneficial in the use of semi-structured diagnostic interview techniques.* |  |  |
|  |  |  | *C11. It may classify clinically normal conditions as psychiatric diagnoses.* |  |  |
|  |  |  | *C12. It may fail to recognize certain psychiatric diagnoses and lead to missed diagnoses.* |  |  |
|  | **Perceived Dehumanization** |  | *C13. Patients and their families may feel uncomfortable with the delegation of their concerns to an artificial intelligence system, perceiving it as a sign that they are not worthy of human attention.* |  |  |
|  | **Treatment** | **Psychoeducation** | *C14. Clinicians often hinder patients' understanding by overusing technical terms. ChatGPT can be used more effectively in this regard.* |  |  |
|  |  | **Structured Psychotherapeutic Support** | *C15. It can be used for skills training and psychoeducation for patients and their families within the framework of Cognitive Behavioral Therapy (CBT).* |  |  |
|  |  |  | *C16. It may demonstrate sufficient creativity in therapy, considering abilities such as storytelling and the use of metaphors.* |  |  |
|  |  |  | *C17. It may assist in the development and implementation of hierarchical exposure tasks in therapeutic settings.* |  |  |
|  |  |  | *C18. It can be used as an aid in assigning behavioral tasks.* |  |  |
| D. Please indicate the appropriate option for each of the following statements regarding your views on the use of ChatGPT-4o by child and adolescents and their families | | | | | |
| **As a Patient-Facing Tool** | **Psychoeducational Support for Families** |  | *D1. ChatGPT-4o may serve as a tool to offer guidance and support to parents and caregivers in the management of their children’s mental health.* |  |  |
|  |  |  | *D2. Cases and their families should consult such tools before seeing a mental health professional.* |  |  |
|  |  |  | *D3. Such conversational agents can help children, adolescents, and their families better prepare for consultations with psychiatrists or psychologists.* |  |  |
|  |  |  | *D4. Children, adolescents, and their families should have access to relevant, accessible, and timely information regarding mental health status and treatment options in a pressure-free environment, enabling them to make informed health-related decisions freely* |  |  |
|  | **Digital Access and Personalization in Youth Mental Health** |  | *D5. ChatGPT-4o saves time by being accessible 24/7 and by answering questions repeatedly without fatigue.* |  |  |
|  |  |  | *D6. ChatGPT-4o has the potential to deliver evidence-based treatment suggestions that are customized according to the individual needs of children, adolescents, and their families.* |  |  |
|  |  |  | *D7. Given that children and adolescents have grown up with such technologies, they may be more predisposed to utilizing tools like ChatGPT-4o to manage psychological difficulties.* |  |  |
| E. Please indicate the appropriate option for each of the following statements regarding the potential roles of ChatGPT-4o as a therapist for children and adolescents | | | | | |
| **Acting as a Therapist** | **Therapeutic Alliance** |  | *E1. As a therapist, ChatGPT-4o is neutral and nonjudgmental; some individuals who fear being judged by a human therapist may find it less intimidating to speak with a machine, thereby expressing sensitive issues more comfortably.* |  |  |
|  |  |  | *E2. Given that human therapists may be influenced by factors such as fatigue, personal life experiences, or their current emotional state, ChatGPT-4o may, in certain situations, offer responses that appear more consistently empathetic.* |  |  |
|  | **Self-Help and Behavior Change Interventions** |  | *E3. Consistent with approaches like Cognitive Behavioral Therapy (CBT) that focus on self-guided skill acquisition, ChatGPT-4o may offer a range of resources and interactive tools to help individuals develop psychological competencies and understanding.* |  |  |
|  |  |  | *E4. ChatGPT-4o can provide personalized suggestions to support behavioral activation in cases with depressive disorders.* |  |  |
|  |  |  | *E5. ChatGPT-4o can provide personalized recommendations for anger management.* |  |  |
|  |  |  | *E6. ChatGPT-4o can provide personalized recommendations for sleep hygiene.* |  |  |
|  |  |  | *E7. ChatGPT-4o can provide personalized recommendations to help reduce anxiety levels.* |  |  |
|  | **Crisis Prevention and Safety Planning** |  | *E8. ChatGPT-4o may play a supportive role in preventing self-harm. Users may seek assistance from ChatGPT-4o in developing a safety plan aimed at reducing self-injurious behavior.* |  |  |
| F. Please indicate the appropriate option for each of the following statements regarding your views on potential bias in ChatGPT-4o (i.e., displaying certain tendencies or deviating from the principle of neutrality). | | | | | |
| **Bias** |  |  | *F1. Even in the absence of newly introduced information, existing biased content related to mental health—such as the stigmatization of mental disorders, inaccurate media portrayals, discriminatory language, and flawed data—may be used to train language models, thereby perpetuating these biases across digital platforms.* |  |  |
| G. Please indicate the appropriate option for each of the following statements regarding your general views on ChatGPT-4o | | | | | |
| **General Impressions** |  |  | *G1. These tools offer greater benefits than risks for both cases and their families, as well as for clinicians.* |  |  |
|  |  |  | *G2. It is essential to utilize ChatGPT-4o to enhance the accessibility and quality of mental health services.* |  |  |
|  |  |  | *G3. I believe that gold standard practices and current approaches can be easily integrated into ChatGPT-4o.* |  |  |
|  |  |  | *G4. The use of ChatGPT-4o in clinical practice may contribute to minimizing the rate of medical errors.* |  |  |
|  |  |  | *G5. Relying on ChatGPT-4o in clinical settings may potentially constrain my capacity for independent problem-solving and creative thinking.* |  |  |
| **Willingness to Use ChatGPT-4o** |  |  | *H. How willing are you to use ChatGPT-4o in your clinical practice?* | *Please rate your willingness on a scale from 1 to 10, where 1 indicates the lowest level of willingness and 10 indicates the highest* | |
| I. Below are some potential areas for improvement in ChatGPT-4o related to child and adolescent mental health.  *Please rate the importance of each feature for you on a scale from 1 to 10 (1 = not important at all, 10 = extremely important).* | | | | | |
| **AI Development Priorities** |  |  | *Ethical* | Ethical issues should be addressed through a multidisciplinary approach | |
|  |  |  | *System Oversight* | It is essential that professionals consistently verify and review the content, and that the system is routinely monitored | |
|  |  |  | *AI Training for Professionals* | Mental health professionals should be trained in the use of artificial intelligence software | |
|  |  |  | *Software – Diagnostic & Treatment Support* | Mental health professionals should be trained in the use of artificial intelligence software | |
|  |  |  | *Software – Psychotherapy Support* | The software should be trained to support psychotherapy practices | |

**Scoring Procedure**

Several items in the survey were intentionally phrased to reflect concerns or skepticism, about the clinical use of ChatGPT-4o (e.g., "B1. Cases and their families may experience greater concerns regarding confidentiality"). To maintain interpretive coherence across the dataset, such negatively framed items were reverse-coded prior to analysis. This applied to a consistent set of items for each group: B1–2, C1, D1, F1, and G4 for psychiatrists; and A1, B1–2, C1–2, C11–13, F1, and G5 for psychologists. Through this approach, higher scores uniformly signified more favorable perceptions of ChatGPT-4o, ensuring that subscale means accurately reflected endorsement or acceptance levels. This scoring strategy is consistent with standard practices in digital health attitude research and facilitates clear, comparable interpretation of scale-level results across respondent groups.

**Interpretation of Mean Scores on the Likert Scale**

To facilitate consistent interpretation of participants’ attitudinal responses, mean scores obtained from the 5-point Likert scale were classified according to predefined intervals. This classification scheme—adapted from Boone and Boone (2012) (1)—helps distinguish between strongly negative, negative, neutral, moderately positive, and strongly positive views. The corresponding ranges and their interpretations are presented in Table S4.3 below. This framework has been widely adopted in applied research for analyzing Likert-type data and was used throughout the present study to interpret subscale and item-level means.

**Table S4.3. Interpretation Guide for Mean Scores on a 5-Point Likert Scale
*(Adapted from Boone & Boone, 2012)(1)***

| **Mean Score** | **Range Interpretation** |
| --- | --- |
| 1.00 – 1.79 | Strongly Negative View |
| 1.80 – 2.59 | Negative View |
| 2.60 – 3.39 | Neutral View |
| 3.40 – 4.19 | Moderately Positive View |
| 4.20 – 5.00 | Strongly Positive View |

Note: This interpretation framework helps classify attitudinal responses and is widely used in applied settings for Likert-type data*.*

**Psychometric Assessment**

Cronbach’s alpha coefficients were calculated to assess the internal consistency of each subscale and the overall instrument. Given the structure and purpose of the present survey—focused on measuring clinician attitudes using multiple-item Likert-type subscales—the interpretation of alpha values followed the guidelines proposed by Tavakol and Dennick (2), which are commonly applied in health professions education and psychological measurement contexts. These thresholds allow for a pragmatic classification of reliability levels (e.g., ≥.90 excellent; .80–.89 good; .70–.79 acceptable), particularly in applied settings where the goal is not scale development but evaluation of existing perceptions. The analysis included only items with coded identifiers (e.g., A1, C4, G2), and all calculations were performed separately for each subscale to account for domain-specific coherence. This approach ensures that each subscale’s reliability is evaluated independently, which is critical in multifactorial instruments assessing complex constructs.

**References**

1. Boone Jr HN, Boone DA. Analyzing likert data. The Journal of extension. 2012;50(2):48.

2. Tavakol M, Dennick R. Making sense of Cronbach's alpha. International journal of medical education. 2011;2:53.
