# Supplementary material for: From promise to practice: insights into ChatGPT-4o use in child and adolescent mental health from professionals
Source: Front Psychiatry. 2025 Sep 26;16:1668814. doi: 10.3389/fpsyt.2025.1668814 (PMC12511095; doi:10.3389/fpsyt.2025.1668814)
Supplement: Supplementary file 5 [file DataSheet5.docx]

Supplementary Material-5

As presented in Table S5.1, the psychiatrist group (N = 96) was predominantly composed of individuals aged 31–40 (64.6%), with over half (51.0%) holding specialist doctor titles and the majority employed at state hospitals. Notably, 86.5% of respondents reported prior experience using ChatGPT-4o, with presentation preparation and academic writing cited as the most common use cases. The psychologist group (N = 70) is summarized in Table S5.2. Female participation was markedly high (92.9%), and the age distribution skewed younger compared to the psychiatrist sample. In this group, private practice and school-based roles were more common, reflecting a broader institutional dispersion. While 80% had previously engaged with ChatGPT-4o, usage patterns appeared more evenly distributed across clinical and educational tasks.

**Table S5.1. Participants (psychiatrists) demographics**

| **Variables** |  | ***N*** | **Relative Frequency (%)** |
| --- | --- | --- | --- |
| Age (in years) | 24-30 | 27 | 28.1 |
|  | 31-40 | 62 | 64.6 |
|  | 41-50 | 7 | 7.3 |
| Gender | Male | 27 | 28.1 |
|  | Female | 69 | 71.9 |
| Professional title | Resident Doctor | 22 | 22.9 |
|  | Specialist doctor | 49 | 51.0 |
|  | Assistant Professor | 17 | 17.7 |
|  | Associate Professor | 8 | 8.3 |
| Affiliated Institution | Private Practice | 8 | 8.3 |
|  | State Hospital | 61 | 63.5 |
|  | University Hospital | 27 | 28.1 |
| ChatGPT-4o Experience ( yes) |  | 83 | 86.5 |
| Purpose of Use | Clinical practice | 46 | 47.9 |
|  | Writing academic papers | 53 | 55.2 |
|  | Preparing presentations | 60 | 62.5 |
|  | Administrative tasks | 35 | 36.4 |

**Table S5.2. Participants (psychologists) demographics**

| **Variables** |  | ***N*** | **Relative Frequency (%)** |
| --- | --- | --- | --- |
| Age (in years) | 24-30 | 38 | 54.3 |
|  | 31-40 | 20 | 28.6 |
|  | 41-50 | 12 | 17.1 |
| Gender | Male | 5 | 7.1 |
|  | Female | 65 | 92.9 |
| Professional title | Psychologist | 35 | 50 |
|  | Clinical Psychologist | 33 | 47.1 |
|  | Assistant Professor | 2 | 2.9 |
| Affiliated Institution | Private Practice | 29 | 41.4 |
|  | State Hospital | 14 | 20 |
|  | University Hospital | 4 | 5.7 |
|  | Department of Psychology, University | 4 | 5.7 |
|  | Ministry of Family and Social Services | 2 | 2.9 |
|  | School | 13 | 18.5 |
|  | Rehabilitation Center | 4 | 5.7 |
| ChatGPT-4o Experience (yes) |  | 56 | 80 |
| Purpose of Use | Clinical practice | 28 | 40 |
|  | Writing academic papers | 29 | 41.4 |
|  | Preparing presentations | 27 | 38.5 |
|  | Administrative tasks | 18 | 25.7 |
